# Model-informed precision dosing of vancomycin and amikacin in critically ill neonates: a pre- and post-intervention study

**DOI:** 10.3389/fphar.2026.1985125

**Published:** 2026-10-16

**Authors:** Beyza Kucukyildiz, Merve Kasikci, Irem Iyigun, Hasan Tolga Celik, Karel Allegaert, Nadir Yalcin

**Affiliations:** 1 Department of Clinical Pharmacy, Faculty of Pharmacy, Hacettepe University, Ankara, Türkiye; 2 Department of Biostatistics, Faculty of Medicine, Hacettepe University, Ankara, Türkiye; 3 Division of Neonatology, Department of Child Health and Diseases, Faculty of Medicine, Hacettepe University, Ankara, Türkiye; 4 Department of Pharmaceutical and Pharmacological Sciences, KU Leuven, Leuven, Belgium; 5 Department of Development and Regeneration, KU Leuven, Leuven, Belgium; 6 Child and Youth Institute, KU Leuven, Leuven, Belgium; 7 Department of Clinical Pharmacy, Erasmus MC, Rotterdam, Netherlands

**Keywords:** amikacin, bayesian forecasting, model-informed precision dosing, neonatal intensive care unit, neonates, vancomycin

## Abstract

**Background:**

Optimal antimicrobial dosing in neonates is complicated by extreme developmental pharmacokinetic (PK) variability. Traditional therapeutic drug monitoring (TDM) fails to achieve PK/pharmacodynamic (PK/PD) target attainment in this vulnerable population.

**Objectives:**

To evaluate whether implementation of Bayesian model-informed precision dosing (MIPD) improved PK/PD target attainment and exposure dose adjustment compared with conventional TDM in critically ill neonates.

**Methods:**

This pre- and post-intervention design comparing a historical retrospective cohort with a prospective MIPD validation single-center cohort study included 61 neonates receiving vancomycin or amikacin. An initial observational phase with standard dosing (n = 30) was followed by an intervention phase (n = 31) using Bayesian dose adjustment. Secondary outcomes included acute nephrotoxicity, ototoxicity, infection-related inflammatory biomarkers (ΔCRP and ΔProcalcitonin), illness severity (ΔSNAPPE-II), and the treatment-duration-to-hospital-length-of-stay ratio (DOT/LOS) in NICU.

**Results:**

Regarding posterior model concordance, agreement for vancomycin was limited, as evidenced by an overall mean absolute error (MAE) of 5.47 mg/L. For amikacin, concordance was modest (overall MAE: 8.96 mg/L; median absolute error [MdAE]: 1.31 mg/L), reflecting persistent modeling challenges. For vancomycin, therapeutic target attainment was higher with MIPD than with standard dosing (5/30, 16.7% vs. 1/26, 3.8%). Similarly for amikacin, therapeutic target attainment was higher in the MIPD group (10/27, 37.0% vs. 5/26, 19.2%). In an exploratory secondary analysis, ΔSNAPPE-II differed between the standard dosing and MIPD cohorts (−2.5 vs. 0; p = 0.040), favoring standard dosing; as SNAPPE-II is not validated for longitudinal monitoring and the design was non-randomized, this does not constitute evidence of clinical benefit for either strategy. For the overall combined cohort, DOT/LOS was significantly lower in the MIPD group (p = 0.043). No differences in the rates of acute kidney injury (6.9% vs. 10.0%, p = 1.000) or ototoxicity (8.0% vs. 7.1%, p = 1.000) were detected, although the study was underpowered to rule out clinically important differences.

**Conclusion:**

Bayesian MIPD was associated with numerically but not statistically higher pharmacodynamic target attainment (AUC/MIC and Cmax/MIC) for vancomycin and amikacin, and no improvement in clinical outcomes was demonstrated. While amikacin modeling requires further refinement, adequately powered trials are needed to definitively assess the impact of MIPD on safety and clinical outcomes.

## Highlights

- Vancomycin and amikacin PK/PD target attainment for the treatment of suspected or proven neonatal sepsis is suboptimal in standard NICU care.
- Posterior model concordance was limited for vancomycin and modest for amikacin, reflecting persistent challenges in modelling neonatal pharmacokinetics.
- For the overall combined cohort, MIPD was associated with a lower treatment-duration-to-hospital-length-of-stay (DOT/LOS) ratio without differences in mortality.
- No differences in acute kidney injury (defined by KDIGO criteria) or ototoxicity were detected between the standard dosing and MIPD cohorts, although the study was underpowered to rule out clinically important differences.

## 1 Introduction

Appropriate antimicrobial use in neonatal sepsis is essential for reducing morbidity and mortality (Smits and De Cock, 2018; Bunn et al., 2023). Pathogen distribution differs by onset, with Gram-negative bacteria (e.g., *Escherichia coli*) predominating in early-onset sepsis, while coagulase-negative staphylococci are most common in late-onset sepsis (Russell et al., 2012). Consequently, amikacin and vancomycin are frequently utilized in the neonatal intensive care unit (NICU) both as empiric therapy for suspected sepsis and as targeted treatment for confirmed infections. However, achieving optimal pharmacokinetic/pharmacodynamic (PK/PD) targets for these agents is complicated by pronounced pharmacokinetic variability in neonates, driven by rapid renal maturation, altered body composition, and critical care interventions (Smits et al., 2013; Oyaert et al., 2015; Allegaert et al., 2017; Kontou et al., 2017).

Model-informed precision dosing (MIPD) combines population PK models with therapeutic drug monitoring (TDM) within a Bayesian framework to support individualized dosing by integrating prior population data with patient-specific drug concentration measurements (Euteneuer et al., 2019; Hughes et al., 2023; Minichmayr et al., 2024). This approach enables estimation of the probability of target attainment, simulation of alternative dosing strategies and optimization of dosing from the first dose without requiring steady-state sampling as is recommended in conventional monitoring guidelines (Rybak et al., 2009; Mould and Upton, 2012; Darwich et al., 2021). Given the risk of nephrotoxicity and ototoxicity associated with commonly used agents in the neonatal intensive care unit (NICU), including aminoglycosides and vancomycin, accurate assessment of drug exposure is essential to balance efficacy and safety (Akunne et al., 2022; Velarde-Salcedo et al., 2023). While previous studies have highlighted the potential of MIPD to improve target attainment for vancomycin in neonates, real-world evidence evaluating its impact on both vancomycin and amikacin remains limited (Touw and van den Anker, 2022; Del Valle-Moreno et al., 2023). In our center, these agents are frequently utilized either as monotherapy or concomitantly for suspected sepsis and confirmed bacterial infections. To bridge the gap between theoretical pharmacometrics and bedside application, clinical implementation requires the adoption of externally validated population PK models rather than generating novel ones (Kantasiripitak et al., 2020). In this study, we utilized a specific, commercially available Bayesian clinical decision support tool (InsightRX) because it integrates well-established neonatal models for both vancomycin and amikacin, facilitating seamless operationalization in routine care. Therefore, this study aimed to evaluate the impact of this Bayesian MIPD platform on PK/PD target attainment, exposure dose adjustment, and selected clinical outcomes in critically ill neonates under real-world NICU conditions.

Given the exploratory nature of integrating this software into our unit, a pre- and post-intervention design was chosen over a randomized controlled trial to establish baseline feasibility and initial clinical impact. The primary objective of this study was to compare standard TDM with Bayesian MIPD regarding PK/PD target attainment (AUC/MIC for vancomycin and Cmax/MIC for amikacin) in NICU patients receiving these agents for suspected or proven sepsis. Secondary objectives included the comparative evaluation of illness severity (ΔSNAPPE-II), treatment-duration-to-hospital-length-of-stay ratio (DOT/LOS), inflammatory biomarkers, and safety endpoints (acute kidney injury and ototoxicity) between the two phases.

## 2 Methods

This pre- and post-intervention design comparing a historical retrospective cohort with a prospective MIPD validation single-center cohort study was conducted in a 30-bed NICU of a tertiary referral university children’s hospital between September 2024 and July 2025. Written informed consent was obtained from the parents or legal guardians of prospective phase neonates prior to study inclusion. For the retrospective observational phase, the ethics committee granted a waiver of informed consent for the review of routinely collected clinical data, as no additional intervention, blood sampling or participant contact was involved. No study-related data collection took place before the approval date. The study period consisted of a 6-month observational phase followed by a 6-month intervention phase in which dosing was guided by MIPD including initial dose selection.

During the observational phase, vancomycin and amikacin were initiated according to standard dosing regimens based on static nomograms and population-average dosing tables derived from postmenstrual age (PMA), postnatal age (PNA), gestational age (GA), body weight, and serum creatinine (SCr), in accordance with the Turkish Neonatal Society’s antimicrobial dosing recommendations (Turkish Neonatal Society, 2026). Conventional TDM was performed according to a standardized routine NICU practice for all enrolled patients. For vancomycin, the first sample was typically obtained as a trough concentration. To strictly minimize iatrogenic blood loss, TDM was generally deferred during the initial 48–72 h empiric window and performed only when a definitive clinical decision was made to prolong targeted therapy. Consequently, the first routine TDM sample (T1) was typically drawn between days 4 and 5 of continuous treatment, frequently before a theoretical steady-state was achieved due to dynamic neonatal renal function. The conventional clinical target during standard care was a trough concentration of 10–15 mg/L. Peak vancomycin concentrations were not obtained; therefore, AUC_0–24h_ values were not calculated via traditional peak-trough equations but were strictly estimated using the Bayesian algorithm. In neonates with impaired renal function, the timing of the first sample was individualized, either by delaying it to align with extended dosing intervals or by measuring it earlier to assess potential drug accumulation. Subsequent sampling was performed during follow-up according to the patient’s clinical course and ongoing renal function. For amikacin, routine TDM samples were collected for all patients as a mandatory safety protocol (not solely at clinician discretion) and consisted primarily of trough concentrations. Similar to vancomycin, initial sampling was typically deferred until days 4–5 to avoid unnecessary blood draws in neonates whose empiric therapy might be discontinued early. This delayed sampling aimed to assess drug clearance and prevent accumulation, aiming for a target trough of <5 mg/L. Peak concentrations were not routinely measured; standard care relied exclusively on trough monitoring, while the specific Cmax/MIC target (≥8–10) evaluated in this study was derived via Bayesian extrapolation. In critically ill neonates with severe or dynamically changing renal impairment, sampling was performed earlier (e.g., 18–24 h post-dose) to guide dosing interval extensions. Sampling was not restricted to a theoretical pharmacokinetic steady state (defined as the completion of 3–5 drug half-lives, which is often unpredictable or unachievable during acute kidney injury). Furthermore, to minimize subjective bias, decisions to sample outside the routine days 4–5 window were driven by objective clinical triggers such as sudden changes in serum creatinine, altered urine output, or the initiation of concomitant nephrotoxic agents rather than a predefined study schedule. Dose adjustment decisions in the observational phase were made by the treating NICU physicians in consultation with the clinical pharmacy team, using measured concentrations and renal function. Specifically, dose amounts were adjusted using simple proportional calculations (e.g., standard linear dose-scaling), while dosing intervals were modified based on serum creatinine trends and age-based nomograms. Although we recognize that critically ill neonates frequently exhibit non-linear pharmacokinetic behavior due to dynamic fluid shifts and rapid maturational changes, this conventional approach reflects the standard of care applied in our unit prior to the implementation of Bayesian forecasting.

In contrast, during the intervention phase, dosing was supported by Bayesian model-informed precision dosing (MIPD) using the InsightRX Nova platform (InsightRX, San Francisco, CA, United States). The same routine TDM sampling criteria were maintained including the prompt discontinuation of antimicrobials and cessation of TDM if microbiological cultures returned negative and sepsis was clinically ruled out. However, dosing decisions were additionally informed by Bayesian posterior estimates generated from explicit patient-specific data: demographic (gestational age, postnatal age, birth weight, and current weight), clinical (serum creatinine and concurrent medications), dosing (exact dose amounts, infusion durations, and administration times), and concentration data (exact sampling times and measured drug levels). The first antimicrobial dose was selected using the recommendations generated by InsightRX Nova, a commercial Bayesian clinical decision support platform. This software utilizes externally validated, population-specific pharmacokinetic models derived from relevant neonatal datasets (specifically, the Frymoyer et al. model for vancomycin and the Illamola et al. model for amikacin). Subsequent dose individualization was updated iteratively whenever new TDM or clinical data became available. Specifically, vancomycin dosing was guided by the one-compartment population PK (popPK) model (Frymoyer et al., 2014), which utilizes PMA and SCr as primary covariates for individualized estimation. The Frymoyer model was specifically selected because it was developed and validated in a large neonatal cohort with gestational ages and weights highly comparable to our NICU population. Furthermore, while we recognize that neonatal SCr during the first days of life may predominantly reflect maternal creatinine transfer, the Bayesian framework mitigates this by initially relying on maturational covariates (PMA) and sequentially updating individual clearance estimates as true neonatal SCr trends emerge. For amikacin, dosing was guided by the popPK model (Illamola et al., 2016), which accounts for weight-based scaling and the non-linear maturation of renal function through PNA and SCr. Similar to the vancomycin model, the Illamola model was selected because it was derived from a large, representative cohort of preterm and term neonates. The potential confounding effect of maternal serum creatinine in the first days of life is addressed by the model’s robust reliance on PNA and weight-based maturation functions for initial dosing, which are subsequently refined by the Bayesian prior-posterior update as true neonatal SCr trends emerge. Dose recommendations generated by the software were manually reviewed by the clinical pharmacy team and then communicated to the treating NICU physicians, who made the final prescribing decision.

Serum vancomycin and amikacin concentrations were measured in the hospital clinical laboratory using a validated liquid chromatography-tandem mass spectrometry (LC-MS/MS) method. Measurements were performed on a SCIEX QTRAP 4500 system. The validated lower limit of quantification (LLOQ) was 0.067 mg/L for amikacin (limit of detection 0.02 mg/L; linear range 0.08–50 mg/L) and 0.10 mg/L for vancomycin (limit of detection 0.03 mg/L; linear range 0.1–80 mg/L). Within-day and between-day coefficients of variation were 7.29% and 5.76% at 2.50 mg/L and 1.25% and 2.91% at 10 mg/L for amikacin, and 7.15% and 6.79% at 4.0 mg/L and 5.10% and 4.35% at 12.5 mg/L for vancomycin. Amikacin results below the LLOQ were returned by the clinical laboratory either as 0.00 mg/L (25 first samples) or, in one further sample, as a value lying between the limit of detection and the LLOQ (0.05 mg/L); all were treated as below the quantification limit. Every such observation was entered into the Bayesian platform exactly as reported, and none was imputed (as LOQ/2 or otherwise), rescaled, or excluded from the Bayesian fitting. The lowest quantifiable amikacin concentration observed in the cohort was 0.08 mg/L. No vancomycin sample fell below its LLOQ (lowest measured concentration 0.33 mg/L), so this issue was confined to amikacin. InsightRX Nova performs bedside MAP Bayesian estimation in which each entered concentration is conditioned on as an observed point value under the residual error structure of the selected population model; likelihood-based approaches for censored observations (Beal M3/M4) are estimation-stage methods implemented in population modelling software such as NONMEM and are not available within a bedside MIPD platform of this type. This is methodologically relevant because censored concentration data constrain the ability of the MAP algorithm to update individual clearance and volume of distribution, and the robustness of the amikacin findings to this limitation was therefore evaluated in a sensitivity analysis (Supplementary Table S4). In both study phases, TDM sampling followed routine clinical practice rather than a protocolized pharmacokinetic sampling design. The number and timing of samples therefore varied according to the antimicrobial used, treatment duration, renal function, and clinical status of the neonate. Sampling times were recorded exactly as documented in the clinical system. The exact time of sample collection relative to dose administration was incorporated into the Bayesian analysis to account for variability in dosing and sampling schedules. T1 refers to the first TDM measurement obtained after treatment initiation, and T2 refers to the subsequent routine TDM measurement when available, typically performed approximately 1 week later as part of the unit’s weekly monitoring workflow.

Measured amikacin and vancomycin serum concentrations were compared with individual Bayesian posterior predictions generated after integration of patient-specific concentration data within the InsightRX environment. Population (*a priori*) predictions were not used for comparison; all error metrics were based on individual Bayesian (*a posteriori*) estimates. Because these estimates already incorporate the observed concentration, the resulting metrics quantify posterior model concordance rather than prospective predictive accuracy, and are referred to as posterior model concordance throughout. Individual PK parameters and their evolution throughout the therapy were estimated using the software’s Bayesian algorithm. Diagnoses were established based on clinical signs, laboratory findings, microbiological cultures, and infection biomarkers (CRP and procalcitonin).

### 2.1 Model-informed precision dosing

For each patient, demographic and clinical data (GA, PMA, birth weight, daily weight, and SCr), detailed dosing histories (dose, interval, infusion duration, and administration time), and TDM results were entered into the InsightRX platform. Relevant clinical factors potentially affecting PK, including comorbidities, dialysis or ECMO support, and co-administered medications (e.g., diuretics, vasopressors, cefepime, piperacillin-tazobactam), were also recorded. Because fluid balance changes dynamically in critically ill neonates, cumulative 24-h daily fluid intake (mL/day) was extracted from nursing flowsheets. While not used as a continuous hourly input or an *a priori* covariate in the popPK models, this daily metric was recorded to retrospectively evaluate its association with the Bayesian-estimated individual clearance.

In practical terms, while the patients were enrolled in sequential study phases (one neonate who began standard-dose therapy in February 2025 was the only patient whose treatment period overlapped with the intervention phase), both groups underwent the same standardized routine TDM sampling protocol. The critical difference between the study phases lay in how these measured concentration data were interpreted to inform subsequent dose individualization: conventional static targeting versus Bayesian MIPD posterior estimation. In the observational phase, dose adjustments were performed by treating physicians using standard linear dose-scaling to achieve static reference trough targets (10–15 mg/L for vancomycin; <5 mg/L for amikacin). During this phase, AUC_0–24h_ or Cmax values were not calculated or targeted in real-time to guide clinical care; standard practice relied entirely on trough concentrations. For the analytical purposes of this study only, individual pharmacokinetic parameters (including drug clearance), AUC_0–24h_ and Cmax values for the observational phase were calculated retrospectively using the same Bayesian MAP algorithm to allow for direct comparative evaluation against the intervention phase. In the intervention phase, the same concentration data were interpreted within a Bayesian framework to estimate individual PK parameters, predict exposure, and simulate alternative dosing regimens. This allowed dose adaptation before or without steady-state conditions, provided that sufficient clinical and concentration data were available.

The platform applied a Maximum A Posteriori (MAP) Bayesian algorithm to integrate observed drug concentrations with established population models for vancomycin (Frymoyer et al., 2014) and amikacin (Illamola et al., 2016), enabling estimation of individual PK parameters (e.g., clearance and volume of distribution) and simulation of optimized dosing regimens targeting predefined PD goals. The overall study design and dosing workflow are summarized in Figure 1. For each sampling event, the model-predicted concentration was extracted at the exact corresponding sampling time recorded in the clinical system to enable direct comparison with the measured value. These predictions correspond to Bayesian posterior (individualized) estimates.

**FIGURE 1:**
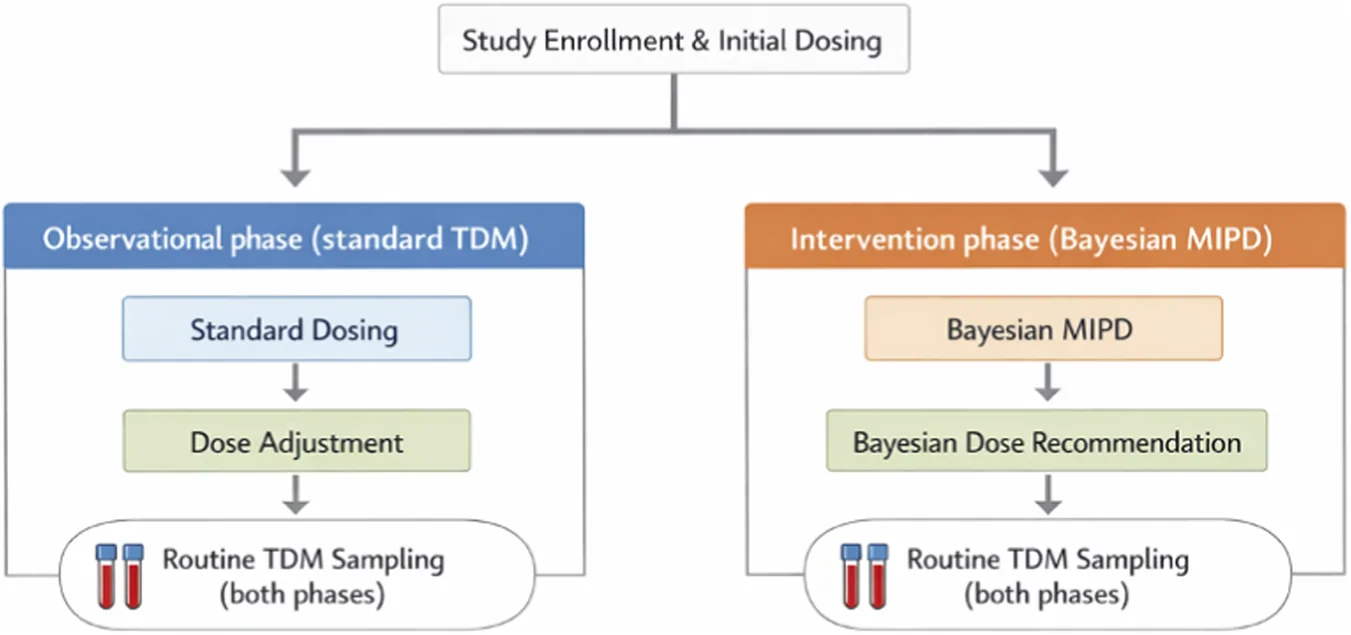
Study design and workflow. During the observational phase, neonates received standard dosing and dose adjustments were performed using conventional TDM and linear dose scaling. During the intervention phase, dosing was supported by Bayesian MIPD using the InsightRX platform. In both phases, routine TDM samples were collected according to clinical practice, but only in the intervention phase were these data used within a Bayesian framework to guide dose individualization.

Target drug exposure for vancomycin was primarily defined as an AUC_0–24h_/MIC ratio ≥400. For all patients, AUC_0–24h_ was mathematically estimated using the Bayesian MAP algorithm within the InsightRX platform, which integrated the exact administered doses, dosing intervals, and available routine TDM samples (predominantly a single trough concentration). During the observational phase, dose adjustments were performed conventionally using linear scaling to achieve trough concentrations of 10–15 mg/L, and AUC_0–24h_ was evaluated retrospectively for analytical comparison only, as real-time bedside AUC calculation using traditional pharmacokinetic equations requires multiple blood samples (e.g., both peak and trough), which were strictly avoided in standard care to minimize iatrogenic blood loss prior to the availability of the Bayesian software. In the intervention phase, the Bayesian platform’s simulation module was used prospectively to adjust doses and directly target the AUC/MIC ≥400 threshold. While current consensus guidelines generally advocate for an AUC_0–24h_ target of 400–600 mg h/L to maximize efficacy and minimize toxicity, the precise upper safety limit in neonates is less definitively established (Rybak et al., 2020). In our study, an AUC_0–24h_ value > 700 mg h/L was strictly defined as the supratherapeutic threshold. This aligns with pediatric evidence indicating that the risk of acute kidney injury significantly increases at absolute exposures exceeding 700–800 mg h/L (Le et al., 2015), while acknowledging that a definitive neonatal-specific toxicity threshold has not yet been established. For amikacin, target exposure was defined as a Cmax/MIC ratio ≥8–10, with a target minimum concentration <5 mg/L (De Cock et al., 2012; Dao et al., 2020; van der Veer et al., 2025). Operationally, exposures were classified as subtherapeutic at Cmax/MIC <8, therapeutic at 8 to <11, and supratherapeutic at ≥11. In the observational phase, standard empiric amikacin dosing typically consisted of 15 mg/kg administered every 24–48 h according to national neonatal guidelines. In the intervention phase, both the initial dose amount and interval were selected prospectively by the Bayesian software. Because peak concentrations were not routinely measured in clinical practice, Cmax values for all patients were mathematically extrapolated using the Bayesian MAP algorithm, which integrated the exact administered dose and the available trough samples. For vancomycin, AUC_0–24h_ values were estimated at two clinically relevant time points for comparative analysis in both study phases. Importantly, AUC_0–24h_ values in the observational phase were derived retrospectively for analytical comparison only and did not influence real-time dosing decisions, whereas in the intervention phase these estimates were available prospectively within the Bayesian workflow. ‘AUC_0–24h_ before dose adjustment’ refers to the estimated 24-h exposure under the initial dosing regimen before the first dose adjustment based on TDM. ‘AUC_0–24h_ after dose adjustment’ refers to the estimated 24-h exposure under the revised dosing regimen after the first dose adjustment. In the observational phase, these AUC_0–24h_ values were retrospectively estimated using the same Bayesian platform and the available patient-specific dosing, sampling, and concentration data, but these estimates were not used to guide clinical care, as this cohort was intended to establish a historical standard-of-care baseline where real-time dosing was strictly driven by conventional trough targets. In the intervention phase, AUC_0–24h_ values were estimated prospectively within the MIPD workflow and were directly used to support dose adjustment. To ensure objective comparison and prevent terminology bias, the terms ‘before dose adjustment’ and ‘after dose adjustment’ are used to strictly describe the timing of the clinical intervention relative to the first TDM result (T1). Accordingly, AUC_0–24h_ before the first dose adjustment represents the expected exposure under the empiric starting regimen. In contrast, AUC_0–24h_ after the first dose adjustment represents the expected exposure under the revised regimen. This uniform definition allows us to impartially compare how effectively conventional TDM versus Bayesian MIPD guided the initial clinical decision, without implying that either method definitively ‘optimized’ the therapy. Post-adjustment exposure estimates were generated only for the subset of intervention-phase neonates who continued therapy and for whom a revised dose was actively simulated and prescribed (vancomycin n = 11; amikacin n = 8), excluding those whose therapy was discontinued after negative cultures. Although doses were also adjusted in the observational phase using conventional linear scaling, Bayesian simulations of exposure under those revised regimens were not generated, so post-adjustment attainment is reported for the intervention phase only.

For vancomycin and amikacin, PD parameters (AUC/MIC and Cmax/MIC) were calculated using exact, patient-specific MIC values whenever provided by the hospital microbiology laboratory. In culture-positive cases where only categorical susceptibility (e.g., ‘Susceptible’) was reported without a discrete MIC value, the epidemiological cutoff (ECOFF) MIC value corresponding to the identified pathogen was applied. In patients without microbiological pathogen growth, default empiric MIC values were assumed in accordance with the literature and EUCAST recommendations (1 mg/L for vancomycin and 4 mg/L for amikacin) to enable the calculation of target PK/PD indices in cases of culture-negative sepsis (defined as the presence of clinical and laboratory signs of infection despite sterile blood and/or cerebrospinal fluid cultures) (European Committee on Antimicrobial Susceptibility Testing, 2025). Because empiric MIC values were applied to 48 of 56 vancomycin evaluations (85.7%) and 50 of 53 amikacin evaluations (94.3%), a sensitivity analysis of target attainment across alternative MIC assumptions (vancomycin 0.5, 1 and 2 mg/L; amikacin 2, 4 and 8 mg/L), holding pathogen-specific ECOFF values constant, was performed (Supplementary Table S5). If a pathogen exhibited *in vitro* resistance, the targeted antimicrobial was discontinued or modified by the treating physicians, and the PK/PD evaluation for that specific agent was concluded at the time of discontinuation. Furthermore, for neonates receiving empiric combination therapy (e.g., amikacin with ampicillin), the target PK/PD indices for vancomycin and amikacin were modeled and evaluated independently of the co-administered agents. All software-based dose recommendations were reviewed before use to ensure patient safety. Maximum dose limits were verified according to established neonatal dosing references and international guidelines for vancomycin and aminoglycosides (Uptodate Lexidrug, 2026a; Uptodate Lexidrug, 2026b).

Posterior model concordance analyses were performed as secondary exploratory assessments. Separately, a categorical model fit classification was generated using the platform’s built-in assessment tool; this is a distinct proprietary categorical output rather than an error metric. According to the platform’s underlying probabilistic methodology, these categories evaluate the statistical likelihood of the measured concentrations occurring within the expected distribution of the selected population model. Specifically, an ‘intermediate’ or ‘poor’ fit indicates that the measured concentrations are statistically ‘unlikely’ or ‘very unlikely’, respectively. Such classifications typically prompt greater clinical scrutiny for potential pre-analytical errors (e.g., incorrect sampling times) or significant physiological deviations (Ekkelboom et al., 2025). As the underlying thresholds are proprietary and not externally defined, these categories were used only as descriptive indicators of model concordance and were not treated as primary analytical outcomes. This approach provided patient-level information on the agreement between observed and predicted concentrations (Konig et al., 2025).

### 2.2 Study population and inclusion/exclusion criteria

Measured drug concentrations were compared with model-based predicted concentrations in neonates who met the study inclusion criteria. Neonates admitted to the NICU during the defined observational and intervention periods, who received at least one of vancomycin or amikacin, and who had at least one measured drug concentration during therapy were included in the study. For the prospective intervention phase, written informed consent from the parents or legal guardians was an additional inclusion requirement. Neonates were excluded if they were discharged or died within the first 24 h after initiation of therapy, if discharge or death occurred before drug concentration measurement. In addition, two neonates with a body weight below 1 kg at the time of treatment initiation were excluded as a precaution during the initial implementation phase, to reduce model instability while the workflow was being established. This was not a blanket weight-based exclusion: the final cohort included four neonates weighing <1 kg at the time of treatment initiation (lowest 795 g) and six with a birth weight <1 kg (lowest 630 g). The two excluded neonates were not left unmonitored; they were managed outside the study protocol using our standard institutional care (static nomograms and conventional TDM with linear dose scaling). Neonates receiving extracorporeal support were not systematically excluded. In terms of PK evaluation, the presence of these circuits was recorded specifically to contextualize anomalous Bayesian posterior estimates such as profound increases in volume of distribution or drug clearance recognizing that the utilized population PK models were not explicitly derived from such complex subpopulations. Regarding clinical and safety outcomes, these patients were analyzed alongside the general cohort rather than as a separate subgroup due to the overall limited sample size. We acknowledge that the inclusion of their clinical outcomes within the broader cohort represents a limitation given their uniquely severe hemodynamic and fluid trajectories.

### 2.3 Outcomes

To assess the clinical course and treatment process, postnatal clinical data, including LOS, treatment duration and the SNAPPE-II score, a validated predictor of disease progression and mortality, were collected. Higher SNAPPE-II scores indicate greater disease severity and mortality risk, whereas lower scores reflect better clinical stability (Richardson et al., 2001). Baseline SNAPPE-II scores were calculated for all patients at the initiation of the targeted antimicrobial therapy. Follow-up scores were uniformly evaluated at the end of the antimicrobial treatment course. ΔSNAPPE-II was calculated as the difference between these follow-up and baseline scores; negative values indicate a decrease in illness severity (clinical improvement), whereas positive values indicate worsening clinical status. Laboratory parameters of inflammation and renal function (CRP, procalcitonin, and SCr), and the presence of acute kidney injury (AKI) were analyzed. Differences in biomarkers were calculated as the absolute change between baseline (at treatment initiation) and the last available measurement during therapy (Δ = final value − baseline value). Measured drug plasma concentrations (mg/L) were compared with model-predicted concentrations to explore the relationship between drug exposure and clinical response, and agreement was descriptively classified as good, intermediate, or poor using the platform-defined categories. Brainstem evoked response audiometry (BERA) was performed as part of routine neonatal hearing screening prior to discharge, and results were evaluated for ototoxicity assessment. AKI was defined using only the serum creatinine component of the KDIGO criteria, as urine output data were not consistently available (Khwaja, 2012). AKI was assessed longitudinally based on changes in SCr values during hospitalization.

### 2.4 Statistical analysis

Due to the absence of prior comparable neonatal MIPD outcome studies, a formal *a priori* sample size calculation was not performed. The study was therefore designed as an exploratory before-after analysis comparing a retrospective observational cohort with a prospectively enrolled intervention cohort, including all eligible patients during the predefined study period.

Data were analyzed using IBM SPSS Statistics version 23. Patients in the observation (standard dosing) and intervention (MIPD) groups were compared primarily in terms of PK/PD target attainment, treatment-related outcomes, selected laboratory parameters, and safety outcomes, while posterior model concordance metrics were included to describe the agreement between observed concentrations and individual Bayesian estimates. Qualitative variables were summarized as frequencies and percentages, whereas quantitative variables were summarized as median (Q1-Q3). Non-normally distributed quantitative variables (e.g., length of stay, treatment duration, ΔSNAPPE-II, and biomarker changes) were analyzed using the Mann-Whitney U test. The relationship between two quantitative variables (e.g., estimated drug clearance and daily fluid intake) was evaluated using Spearman’s correlation coefficient. The association between two categorical variables (e.g., PK/PD target attainment categories, mortality, acute kidney injury, and ototoxicity) was evaluated using Pearson chi-square test, Fisher’s exact test, or Fisher-Freeman-Halton test, as appropriate, taking into account the dimensions of the cross tables and the expected frequencies.

Statistical significance was defined as p < 0.05. MAP Bayesian fitting was performed via the InsightRX platform to individualize the vancomycin (Frymoyer et al., 2014) and amikacin (Illamola et al., 2016) models for each patient. Posterior model concordance analyses were performed as secondary exploratory assessments to contextualize the pharmacometric behavior of the implemented models. Because the underlying neonatal physiological and maturational factors governing drug disposition do not differ systematically between the observational and intervention timeframes, posterior model concordance was evaluated on the overall pooled cohort rather than being stratified by study phase. These metrics describe the concordance between observed and model-estimated concentrations rather than the clinical impact of the MIPD intervention. These analyses were not treated as primary comparative endpoints, but rather as supportive metrics for interpreting exposure estimation and dose adjustment performance. Posterior model concordance was quantified using standard error metrics, the details and results of which are provided in the Supplementary Appendix. Because a substantial proportion of first amikacin samples were below the LLOQ, an additional sensitivity analysis was performed in which the amikacin Cmax/MIC target attainment comparison was repeated after restricting the cohort to neonates with a quantifiable T1 concentration; in this reduced subset the Fisher-Freeman-Halton exact test was applied because expected cell frequencies fell below five. Because Cmax was extrapolated rather than measured, the robustness of the amikacin Cmax/MIC classification was additionally examined by quantifying each neonate’s relative distance from the nearest classification boundary and by recalculating the classification under systematic multiplicative error in the estimated Cmax (factors 0.7–1.3). These perturbation magnitudes were illustrative bounds rather than estimates derived from the model’s error structure, as the platform does not export posterior standard errors for individual parameters.

Target attainment for AUC/MIC and Cmax/MIC was visualized using heatmap plots stratified by study group, postnatal age categories (0–14, 15–30, and >30 days), and categories of body weight at treatment initiation (<2000 g, 2000–2800 g, and >2800 g). The number of patients contributing to each stratum is displayed within the corresponding cell. Several strata contain few patients; these stratified analyses are therefore descriptive only and no inferential testing was performed. These specific stratification cutoffs were selected *a priori* based on standard neonatal antimicrobial dosing guidelines (including the Turkish Neonatal Society recommendations and UpToDate Lexidrug) (Turkish Neonatal Society, 2026; Uptodate Lexidrug, 2026a; Uptodate Lexidrug, 2026b). These references traditionally utilize these specific postnatal age and weight thresholds to categorize expected phases of renal maturation and to guide corresponding empirical dosing intervals in clinical practice. Heatmap visualizations were generated using the ggplot2 package in R software (Wickham, 2016). These stratification categories were predefined to reflect clinically relevant subgroups in neonatal PK based on maturational changes in renal function.

## 3 Results

A total of 61 patients who met the explicit inclusion criteria (detailed in the Study Population subsection) were enrolled in the study among 64 patients admitted to the NICU, who were treated with at least one of the drugs vancomycin or amikacin and received routine TDM throughout the treatment period. Specifically, among the 61 enrolled neonates, 56 received vancomycin (26 in the standard dosing group; 30 in the MIPD group), and 53 received amikacin (26 in the standard dosing group; 27 in the MIPD group). Consequently, a large proportion of the cohort (n = 48) received concomitant therapy with both agents. As detailed in the Methods section, a formal *a priori* sample size calculation was not performed due to the absence of prior comparable neonatal MIPD studies, reflecting the exploratory design of this cohort. Furthermore, for patients receiving concomitant nephrotoxic agents (e.g., concurrent vancomycin and amikacin therapy), any subsequent impact on renal function was continuously captured via serial serum creatinine monitoring and automatically integrated as a dynamic clinical variable into the Bayesian posterior clearance estimates. One patient was excluded due to death before serum drug level measurement at admission (1.5%), and two due to a body weight below 1 kg at treatment initiation (3.1%).

Baseline demographic and clinical characteristics stratified by study group are presented in Table 1.

**TABLE 1:** Baseline demographic and clinical characteristics stratified by study group (n = 61).

General characteristics	Standard (n = 30)	MIPD (n = 31)	p-value
Sex, male, n (%)	18 (60.0%)	21 (67.7%)	0.529
Cesarean delivery, n (%)	25 (83.3%)	25 (80.6%)	0.785
Twin birth, n (%)	5 (16.7%)	4 (12.9%)	0.731
APGAR score at 1 min, median (IQR) (n = 56)	8 (7–8)	7.5 (6–9)	0.946
APGAR score at 5 min, median (IQR) (n = 54)	9 (8–9)	9 (8–10)	0.678
APGAR score at 10 min, median (IQR) (n = 34)	10 (9–10)	10 (9–10)	0.938
Gestational age (weeks), median (IQR)	35 (32–37)	35 (31–38)	0.971
Birth weight (g), median (IQR)	2435 (1500–2905)	2850 (1100–3140)	0.399
Postnatal age at treatment initiation (days), median (IQR)	10.5 (5–24)	13 (3–34)	0.603
Extracorporeal therapy (dialysis), n (%)	1 (3.3%)	3 (9.7%)	0.612
SNAPPE-II score at treatment initiation, median (IQR)	9 (0–19)	5 (0–18)	0.811
Baseline serum creatinine (mg/dL), median (IQR)	0.44 (0.28–0.70)	0.56 (0.34–0.80)	0.217

APGAR, Appearance-Pulse-Grimace-Activity-Respirations; IQR, interquartile range.

Regarding the primary study outcomes, prior to evaluating PK/PD target attainment, sampling characteristics and dosing regimens were summarized. For vancomycin, 56 initial samples (T1) were obtained (Standard: n = 26; MIPD: n = 30) at a median time of 108.0 (IQR, 78.0–162.0) hours after antibiotic initiation in the standard group and 120.0 (IQR, 72.0–168.0) hours in the MIPD group. Only six follow-up samples (T2) were obtained (Standard: n = 4; MIPD: n = 2). For amikacin, 53 initial samples (T1) were obtained (Standard: n = 26; MIPD: n = 27) at a median time of 132.0 (IQR, 96.0–162.0) and 120.0 (IQR, 72.0–168.0) hours, respectively, with five follow-up samples (T2) (Standard: n = 3; MIPD: n = 2). Among these, 26 of the 53 initial T1 samples (49.1%) and one of the 5 T2 samples fell below the quantification limit. The low number of repeat (T2) samples was a direct consequence of the relatively short remaining treatment durations after T1 (overall median therapy was 8–9.5 days); if clinical improvement and stable renal function were observed following the initial adjustment, further pharmacokinetic sampling was intentionally avoided to prevent iatrogenic anemia.

Regarding dosing, the median initial vancomycin dose administered before adjustment was 30.2 (IQR, 25.5–31.8) mg/kg/dose in the standard group and 25.7 (IQR, 19.9–29.7) mg/kg/dose in the MIPD group. After the first TDM-informed adjustment, which was performed for all patients based on the initial T1 concentrations, the revised median doses were 30.9 (IQR, 30.1–31.7) and 42.9 (IQR, 36.9–48.8) mg/kg/dose, respectively. For amikacin, the median initial doses were 15.0 (IQR, 14.4–15.6) and 14.3 (IQR, 13.4–15.1) mg/kg/dose, respectively, which were adjusted to 17.7 (IQR, 14.0–21.3) mg/kg/dose in the MIPD group (no subsequent amikacin dose modifications were recorded in the standard group; the three repeat T2 samples in this group were obtained strictly for safety monitoring to rule out drug accumulation during prolonged therapy).

Consequently, the actual median estimated AUC/MIC for vancomycin prior to the first dose adjustment was 247.5 (IQR, 191.0–283.5) in the standard group and 231.0 (IQR, 167.8–393.0) in the MIPD group. For amikacin, the actual median estimated Cmax/MIC at T1 was 11.4 (IQR, 6.3–12.8) and 10.4 (IQR, 8.8–12.7), respectively.

Regarding the study outcomes, PK/PD target attainment and selected safety outcomes are presented in Table 2. A numerically higher proportion of patients in the MIPD group achieved therapeutic target levels, but neither comparison reached statistical significance (AUC/MIC, p = 0.068; Cmax/MIC, p = 0.304), and the study was not powered to detect differences of this magnitude. All PK/PD target attainment proportions are drug-specific and were therefore calculated using the number of neonates who actually received the respective agent (vancomycin: 26 standard, 30 MIPD; amikacin: 26 standard, 27 MIPD) rather than the total number of neonates enrolled in each phase (30 and 31, respectively).

**TABLE 2:** PK/PD target attainment and selected safety outcomes in the observational and intervention phases.

Outcome	Exposure category	Standard (n = 30)	MIPD (n = 31)	p-value
AUC/MIC before first dose adjustment (vancomycin; standard n = 26, MIPD n = 30)	Therapeutic	1/26 (3.8%)	5/30 (16.7%)	0.068
	Subtherapeutic	25/26 (96.2%)	22/30 (73.3%)	
	Supratherapeutic	0/26 (0%)	3/30 (10.0%)	
AUC/MIC after first dose adjustment (vancomycin; intervention phase only, n = 11; descriptive)	Therapeutic	-	3/11 (27.3%)	-
	Subtherapeutic	-	8/11 (72.7%)	
	Supratherapeutic	-	0/11 (0%)	
Cmax/MIC before first dose adjustment (amikacin; standard n = 26, MIPD n = 27)	Therapeutic	5/26 (19.2%)	10/27 (37.0%)	0.304
	Subtherapeutic	8/26 (30.8%)	5/27 (18.5%)	
	Supratherapeutic	13/26 (50.0%)	12/27 (44.4%)	
Cmax/MIC after first dose adjustment (amikacin; intervention phase only, n = 8; descriptive)	Therapeutic	-	3/8 (37.5%)	-
	Subtherapeutic	-	3/8 (37.5%)	
	Supratherapeutic	-	2/8 (25.0%)	
Mortality		4/30 (13.3%)	2/31 (6.5%)	0.425
Ototoxicity		2/25 (8.0%)	2/28 (7.1%)	1.000
Acute kidney injury (standard n = 29, MIPD n = 30)		2/29 (6.9%)	3/30 (10.0%)	1.000

AUC, area under the concentration–time curve; MIC, minimum inhibitory concentration; MAP, maximum *a posteriori*. Data are n/N (%). Denominators are drug- and assessment-specific and are given in each row label; safety denominators reflect the number of neonates with the corresponding assessment available. 'Before first dose adjustment’ refers to exposure under the initial empiric regimen; ‘after first dose adjustment’ refers to the Bayesian-simulated exposure under the revised regimen. All values are individualized posterior (MAP) estimates incorporating the measured T1 sample. Post-adjustment exposure was simulated only in the intervention phase; doses were also revised in the observational phase, by conventional linear scaling, but no Bayesian simulation of exposure under those regimens was generated. The post-adjustment rows are therefore descriptive of the intervention phase alone and do not permit comparison of dose-adjustment effectiveness between strategies, which is why no p-value is reported. Full definitions are given in Section 2.1.

For interpretability, vancomycin AUC_0–24h_ was evaluated before the first TDM-informed dose revision in both groups; exposure under the revised regimen was simulated in the intervention phase only. No statistically significant differences were detected between groups in mortality, ototoxicity, or AKI; given the low event rates, the study was underpowered to rule out clinically important differences (Table 2). Of the four patients who failed the BERA test, three had received concomitant vancomycin and amikacin therapy.

Heatmap visualizations were used to illustrate the distribution of target exposure ranges (AUC/MIC for vancomycin and Cmax/MIC for amikacin) according to intervention status, postnatal age and body weight at treatment initiation (Figures 2, 3). For vancomycin, at the first sampling point (T1) prior to dose adjustment, one of 26 patients (3.8%) in the standard dosing group and five of 30 patients (16.7%) in the MIPD group achieved the therapeutic range. Most patients remained subtherapeutic (AUC/MIC <400), particularly in the standard dosing group (96.2%), whereas individualized dosing was associated with higher target attainment in the MIPD group, without formal statistical comparison (73.3% subtherapeutic). Subtherapeutic exposure predominated in every postnatal age and weight stratum, without a consistent gradient by age (83.3%, 87.5% and 83.3% for 0–14, 15–30 and >30 days) or by weight (89.5%, 90.0% and 77.8% for <2000 g, 2000–2800 g and >2800 g). These strata are small and the proportions are descriptive only.

**FIGURE 2:**
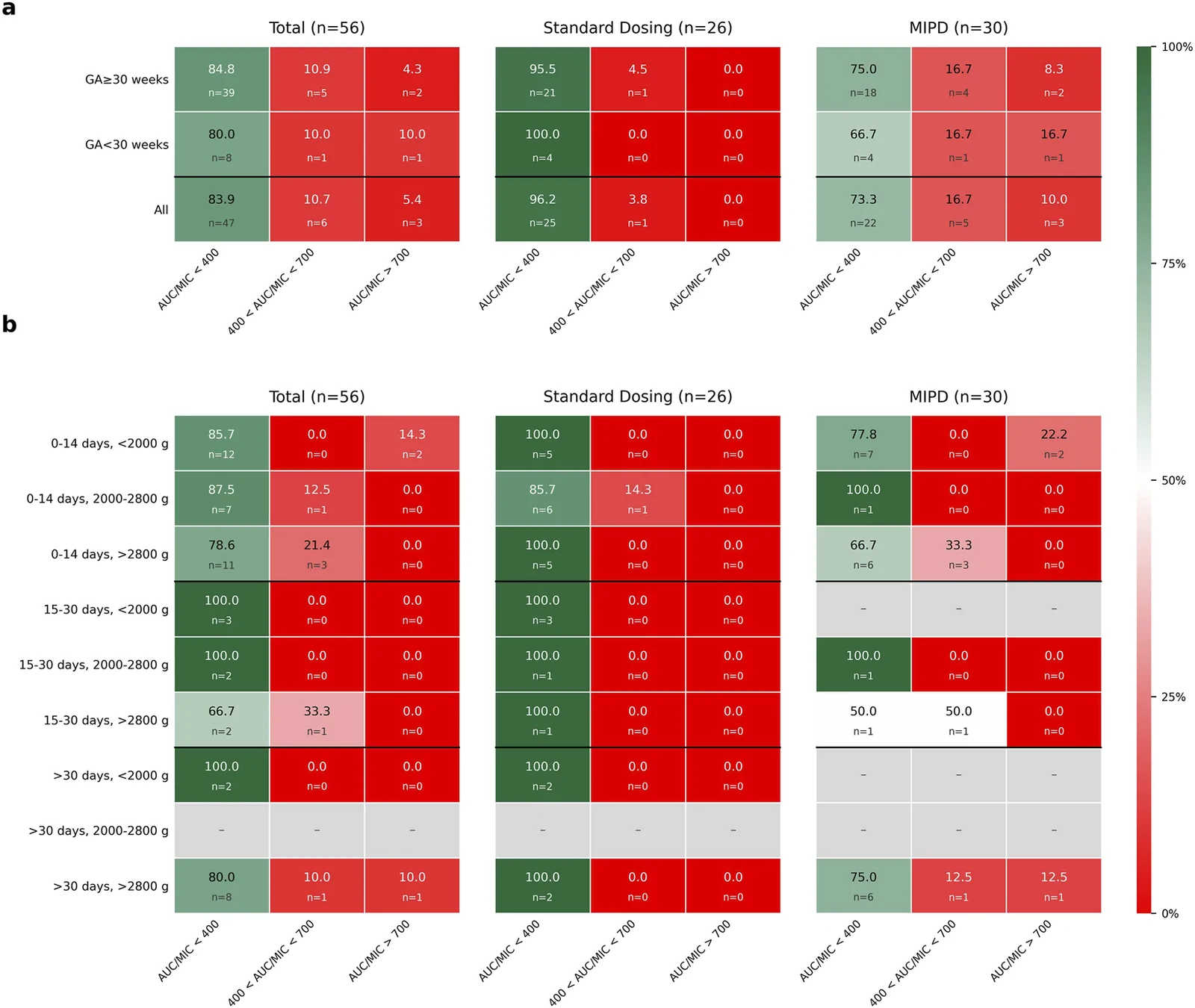
Heatmap visualization of target attainment during vancomycin therapy at the first sampling point (T1). **(a)** Target attainment in the total cohort, standard dosing group and MIPD group, stratified by gestational age. **(b)** Target attainment stratified by postnatal age at treatment initiation and body weight at treatment initiation. The number of patients is shown in each cell; several strata contain few patients and these panels are descriptive only, with no inferential testing performed. Strata containing no patients are shown in grey. T1 = first sampling point; AUC = area under the concentration-time curve; MIC = minimum inhibitory concentration; GA = gestational age. Note: The color gradient (from red to green) strictly represents the relative frequency distribution (0%–100%) within each subgroup and does not imply the clinical desirability, efficacy, or safety of the respective target attainment categories.

**FIGURE 3:**
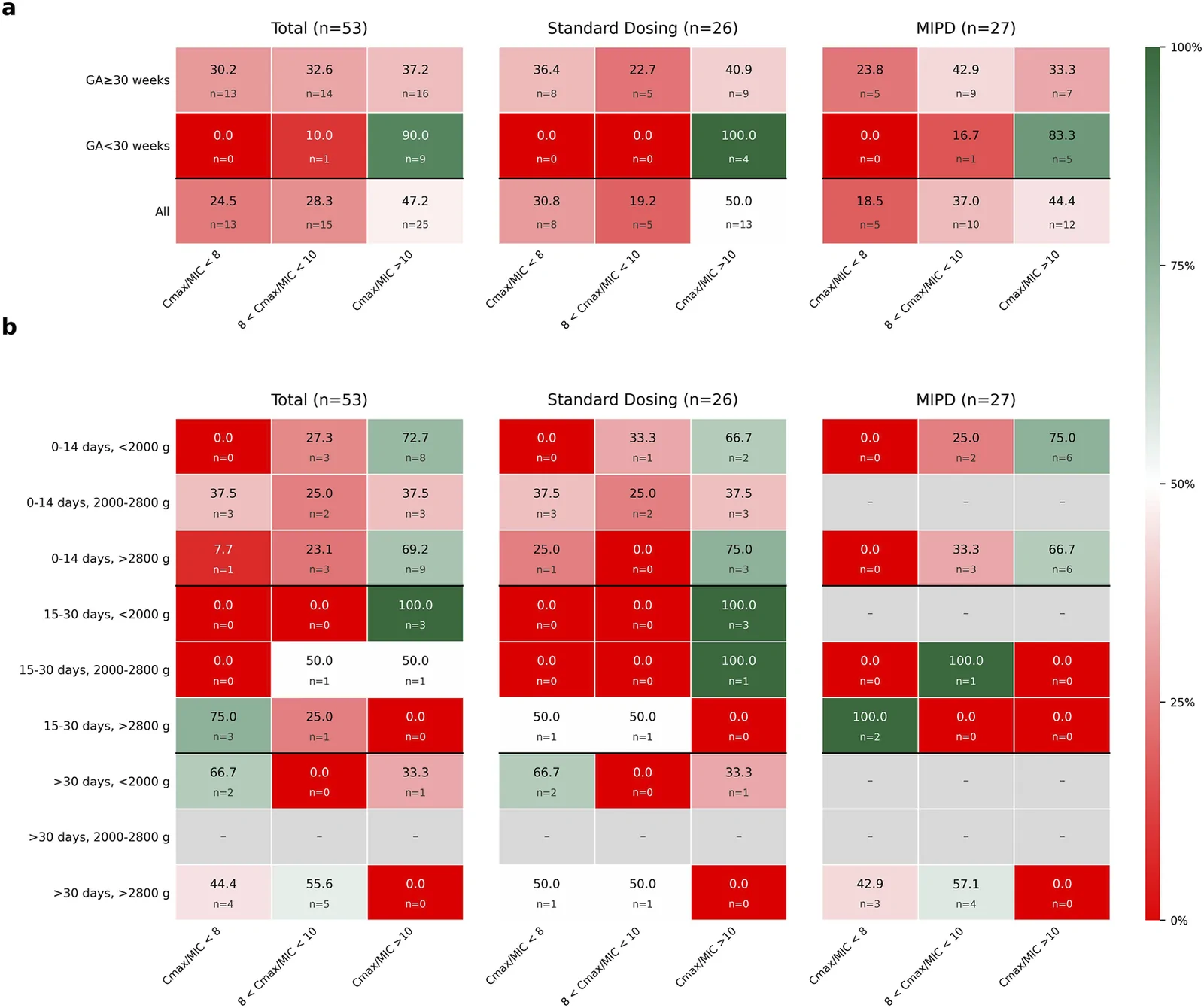
Heatmap visualization of target attainment during amikacin therapy at the first sampling point (T1). **(a)** Target attainment in the total cohort, standard dosing group and MIPD group, stratified by gestational age. **(b)** Target attainment stratified by postnatal age at treatment initiation and body weight at treatment initiation. The number of patients is shown in each cell; several strata contain few patients and these panels are descriptive only, with no inferential testing performed. Strata containing no patients are shown in grey. T1 = first sampling point; Cmax = maximum concentration; MIC = minimum inhibitory concentration; GA = gestational age. Note: The color gradient (from red to green) strictly represents the relative frequency distribution (0%–100%) within each subgroup and does not imply the clinical desirability, efficacy, or safety of the respective target attainment categories.

According to the amikacin heatmap, approximately half of the overall population achieved Cmax/MIC values > 10, indicating that amikacin exposure generally exceeded the therapeutic target. High rates of Cmax/MIC >10 were most commonly observed in infants aged 0–14 days and in those weighing <2000 g. In contrast, an increased proportion of Cmax/MIC <8 was observed among infants with a PNA >30 days, suggesting suboptimal dosing due to increased renal clearance; the numbers within these strata were, however, very small. In the intervention group, lower subtherapeutic rates were observed; however, no formal statistical comparison was performed for these stratified analyses (Figure 3). In a sensitivity analysis restricted to the 27 neonates with a quantifiable T1 amikacin concentration (standard dosing, n = 15; MIPD, n = 12), therapeutic Cmax/MIC attainment was 3/15 (20.0%) versus 5/12 (41.7%) (p = 0.599), consistent in direction with the full cohort. The proportion of samples below the quantification limit did not differ significantly between groups (standard dosing 11/26, 42.3%; MIPD 15/27, 55.6%; p = 0.414), and none of the 56 vancomycin samples fell below its LLOQ (Supplementary Table S4). Because Cmax was derived by extrapolation, we also quantified the robustness of this classification. The median distance from the nearest classification boundary was 14.6% of the estimated Cmax/MIC value, with 18 of 53 neonates (34.0%) lying within 10% of a boundary. A systematic 10% error in the estimated Cmax would reclassify 7 to 11 of 53 neonates (13.2%–20.8%) and a 20% error would reclassify 11 to 22 (20.8%–41.5%); the direction of the between-group difference was preserved for multiplicative errors between 0.8 and 1.2 but reversed at 0.7 and 1.3 (Supplementary Table S6).

The platform-defined model fit was good in six of 52 evaluable amikacin fits (11.5%), intermediate in 21 (40.4%) and poor in 25 (48.1%); for vancomycin the corresponding figures were 18 of 56 (32.1%), 18 (32.1%) and 20 (35.7%). Among the 26 neonates with a first amikacin sample below the quantification limit, the posterior-predicted concentration was bimodal: it was below 2 mg/L in 13 (median 0.83 mg/L) but remained at 9 mg/L or above in 13 (median 15.2 mg/L, maximum 29.7 mg/L). This division corresponded exactly to the platform fit classification, all 13 discordant cases being classified as poor fit. The discordant subgroup had higher estimated exposure (median Cmax/MIC 12.5 vs. 9.0, p = 0.006) and 10 of 13 were classified as supratherapeutic, accounting for 10 of the 25 supratherapeutic amikacin classifications in the cohort; these neonates were distributed across both phases (5 standard dosing, 8 MIPD).

Target attainment was strongly dependent on the assumed MIC: halving the assumed vancomycin MIC to 0.5 mg/L increased therapeutic attainment from 3.8% to 50.0% under standard dosing and from 16.7% to 40.0% under MIPD, whereas doubling it to 2 mg/L rendered the target unattainable in both groups. The MIPD group was less frequently subtherapeutic at four of the six MIC values examined, the two exceptions being the extreme assumptions at which the target was essentially unattainable in both groups, and in the MIC-independent analysis of absolute exposure an AUC0-24h of at least 400 mg h/L was reached in 8/28 (28.6%) of MIPD neonates versus 1/20 (5.0%) of standard dosing neonates evaluated against the empiric MIC (p = 0.061) (Supplementary Table S5).

Clinical outcomes and exposure-related variables are summarized in Table 3. In an exploratory secondary analysis, ΔSNAPPE-II differed between the two groups (p = 0.040), with a more negative median change in the standard dosing group indicating a greater reduction in illness severity in that group. SNAPPE-II scores at treatment initiation were comparable between groups (Table 1), so this difference cannot be attributed to a measured imbalance in illness severity at baseline. It should nonetheless not be read as evidence of a clinical benefit of either dosing strategy: SNAPPE-II is not validated for longitudinal monitoring, the analysis was exploratory and unadjusted for multiplicity, and the sample size was small. Although the DOT/LOS ratio was significantly lower in the MIPD group (p = 0.043), this finding should be interpreted as an exploratory indicator of improved antimicrobial stewardship rather than a direct clinical benefit. While this ratio suggests that MIPD may contribute to more rational antimicrobial utilization, it does not correlate with a statistically significant difference in total LOS (p = 0.367) or mortality, thus precluding any claims of superior clinical efficacy. Treatment duration refers to periods during which patients received the targeted study antimicrobials (vancomycin and/or amikacin). Overall, 37 neonates (60.7%) received empiric therapy for suspected culture-negative sepsis, while 24 neonates (39.3%) were treated for confirmed culture-positive infections. Among the culture-positive cases, all isolates tested for vancomycin susceptibility (n = 8) were susceptible. For amikacin, three isolates were susceptible and four exhibited *in vitro* resistance; as dictated by our protocol, amikacin was promptly discontinued in these resistant cases and their specific PK/PD evaluations were concluded. Because our institutional automated microbiology systems report susceptibility primarily as threshold inequalities (e.g., ≤1 mg/L) rather than discrete exact values, target attainments for all susceptible isolates were calculated using their respective EUCAST ECOFF values, whereas no MIC denominators were assigned to the resistant cases. Patients who required re-initiation of the targeted study antibiotics during hospitalization had longer treatment durations. Similarly, no significant differences were found in Δprocalcitonin, ΔSCr, or ΔCRP values.

**TABLE 3:** Clinical outcomes and exposure-related variables by study group.

Variables		Standard (n = 30)		MIPD (n = 31)		p-value
		n	Median (Q1-Q3)	n	Median (Q1-Q3)	
ΔSNAPPE-II scores		30	−2.5 (-12 - 0)	31	0 (-5 - 0)	0.040
Treatment duration (days)	Vancomycin	26	8 (7–11)	30	8.5 (6–10)	0.524
	Amikacin	26	9.5 (7–11)	27	8 (6–10)	0.536
LOS (days)		30	33.5 (20–93)	31	51 (28–85)	0.367
Treatment duration-to-LOS ratio (DOT/LOS)		30	0.3 (0.2–0.4)	31	0.2 (0.1–0.3)	**0.043**
Daily fluid intake (mL)		27	240 (169–400)	31	382 (244–486)	**0.024**
Daily fluid intake (mL/kg/day)		27	120.7 (85.7–157.9)	31	147.6 (126.3–197.6)	**0.035**
Estimated clearance (mL/min/1.73m^2^)-Schwartz		30	63 (39–98)	31	48 (30–83)	0.240
ΔSCr		29	−0.1 (−0.30 - 0.01)	30	−0.12 (−0.32–−0.01)	0.958
ΔCRP		26	−0.3 (−3.8 - 4.4)	20	−1 (−55.4–3.7)	0.258
ΔProcalcitonin		6	−0.34 (−0.64–−0.01)	2	−0.03 (−0.17 - 0.11)	-

LOS, length of stay; SCr, serum creatinine; CRP, C-reactive protein; SNAPPE-II, Score for Neonatal Acute Physiology with Perinatal Extension-II. Δ, change calculated as final value − baseline value. Bold p values are statistically significant (p < 0.05).

Analysis of hospital stay length showed that longer hospitalizations were observed in the MIPD group compared with standard care, although the difference was not statistically significant. Although the distribution of vancomycin and amikacin treatment durations did not differ statistically, the distributions appeared visually narrower in the MIPD group (Figure 4).

**FIGURE 4:**
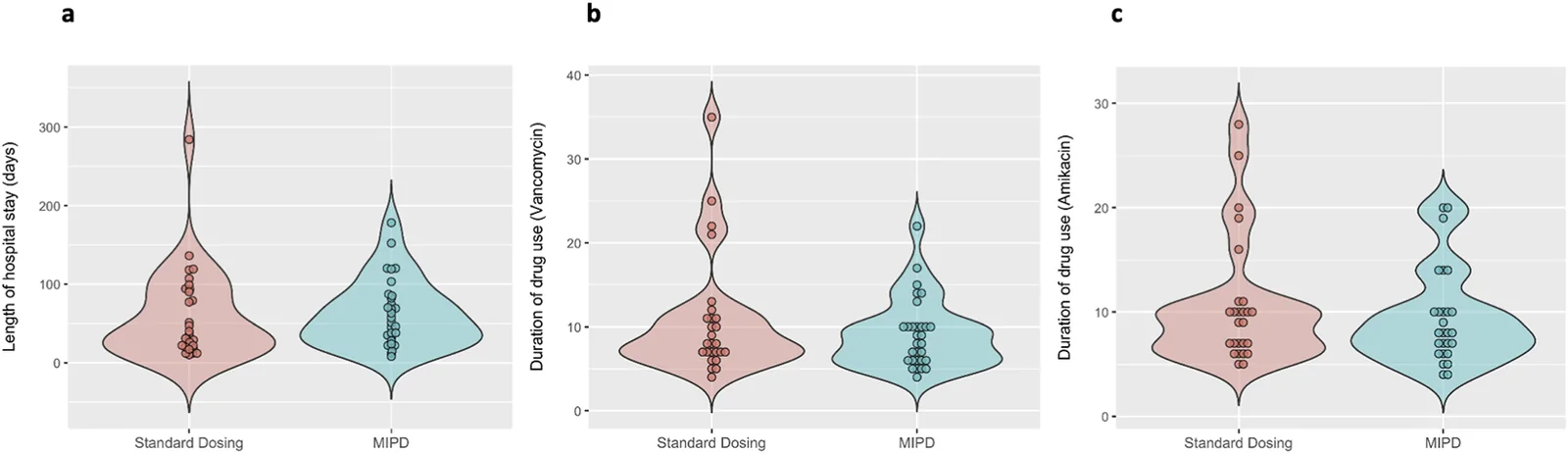
Violin plot with beeswarm of hospital length of stay (days) and treatment durations (days). **(a)** Length of hospital stay (days). **(b)** Duration of vancomycin use (days). **(c)** Duration of amikacin use (days). LOS = length of stay; MIPD = model-informed precision dosing.

### 3.1 Effects of fluid balance and diuretic use on drug clearance

Fluid balance and diuretic therapy are known to influence drug distribution volume and clearance in neonates. Spearman correlation analyses were performed to assess the associations between individual Bayesian-estimated clearance values (final MAP estimates per patient) and ΔSCr and daily fluid intake, stratified by diuretic use. As each patient contributed a single clearance estimate, repeated-measures analysis was not required. The findings are presented in Table 4 and Figure 5. In neonates receiving diuretics (n = 13), daily fluid intake was positively correlated with vancomycin clearance (Spearman’s rho = 0.666, p = 0.013). Among patients not receiving diuretics, daily fluid intake was positively correlated with individual vancomycin clearance (Spearman’s rho = 0.395, p = 0.012) and amikacin clearance (rho = 0.386, p = 0.017).

**TABLE 4:** Correlations between clearance, ΔSCr, and daily fluid intake according to diuretic use.

Diuretic use			Population vancomycin clearance (L/h)	Individual vancomycin clearance (L/h)	Population amikacin clearance (L/h)	Individual amikacin clearance (L/h)
Yes	ΔSCr	Spearman’s rho	−0.366	−0.355	0.339	0.144
		p	0.218	0.233	0.281	0.656
		n	13	13	12	12
	Daily fluid intake (mL)	Spearman’s rho	0.641	0.666	0.482	0.518
		p	**0.018**	**0.013**	0.112	0.084
		n	13	13	12	12
No	ΔSCr	Spearman’s rho	0.43	0.395	0.587	0.601
		p	0.005	0.011	<0.001	<0.001
		n	41	41	39	39
	Daily fluid intake (mL)	Spearman’s rho	0.536	0.395	0.544	0.386
		p	**<0.001**	**0.012**	**<0.001**	**0.017**
		n	40	40	38	38

ΔSCr, change in serum creatinine. Bold p values are statistically significant (p < 0.05).

**FIGURE 5:**
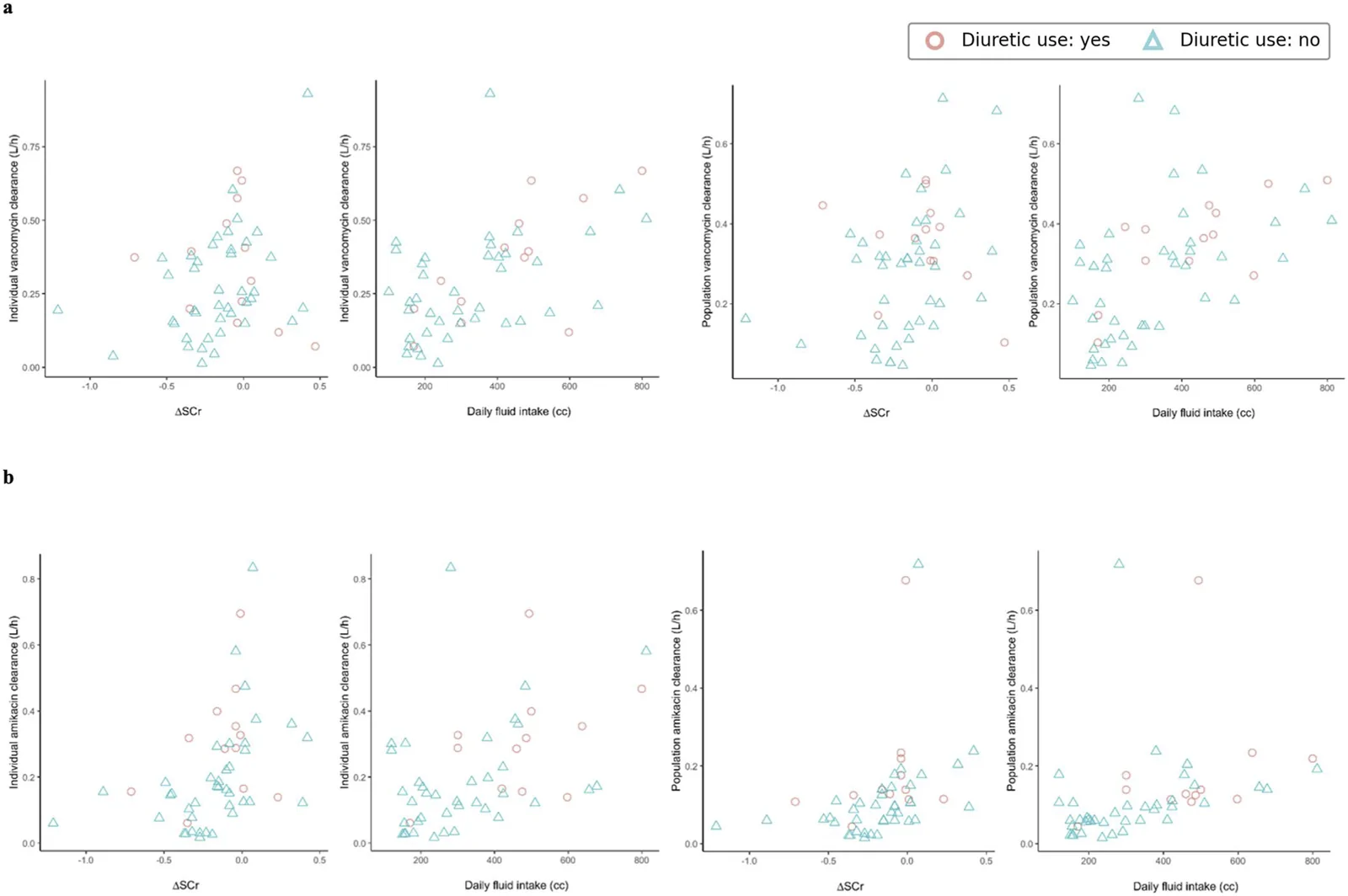
Scatter plots showing the relationships between change in serum creatinine (ΔSCr), daily fluid intake and estimated drug clearance, according to diuretic use. Circles represent neonates receiving diuretics and triangles those not receiving diuretics; symbol definitions are shown within the figure. Numbers of neonates contributing to each panel differ slightly between agents because clearance estimates were not available for every patient (vancomycin: 13 receiving and 41 not receiving diuretics; amikacin: 12 and 39, respectively; Table 4). **(a)** Vancomycin: individual and population clearance versus ΔSCr and daily fluid intake. **(b)** Amikacin: individual and population clearance versus ΔSCr and daily fluid intake. SCr = serum creatinine; CL = clearance; L/h = liters per hour; ΔSCr = change in serum creatinine.

In patients receiving diuretics, a negative but non-significant association between ΔSCr and clearance was observed, indicating a trend toward lower clearance with increasing SCr. In this study, the effects of fluid balance, diuretic use, and changes in SCr on vancomycin and amikacin clearance were evaluated in the overall patient population; however, comparative analyses between the standard and MIPD groups could not be performed due to limited data. The potential relationship between volume of distribution and fluid intake was not assessed and may represent an additional factor influencing drug exposure.

Detailed analyses of posterior model concordance and the corresponding error metrics are provided in the Supplementary Appendix.

## 4 Discussion

Due to marked interindividual pharmacokinetic variability and ongoing organ maturation, achieving optimal antibiotic exposure in neonates remains a major clinical challenge (Hughes et al., 2023). In this context, the present study extends the application of MIPD, which has previously been explored mainly in adult and pediatric populations (Ewoldt et al., 2022; Chai et al., 2023; Velarde-Salcedo et al., 2023; Van Wynsberge et al., 2024), to critically ill neonates in the NICU.

### 4.1 PK and PD considerations

In this study, the main analysis focused on PK/PD target attainment, exposure dose adjustment, and selected clinical outcomes under standard dosing and MIPD strategies. Similar to the randomized controlled DOLPHIN trial conducted by Ewoldt et al. in critically ill adult patients, no statistically significant difference was observed between standard dosing and MIPD groups in terms of PD target attainment (Ewoldt et al., 2022). In the DOLPHIN trial, as in our cohort, a numerically higher proportion of patients in the MIPD group achieved therapeutic exposure. Specifically, therapeutic target attainment for vancomycin (5/30, 16.7% vs. 1/26, 3.8%) and amikacin (10/27, 37.0% vs. 5/26, 19.2%) was higher in the MIPD group compared with standard care. However, it is important to emphasize that the absolute proportion of patients reaching these targets remained well below 50% across all groups. In our study, this trend likewise did not reach statistical significance, likely due to limited sample size and the persistent pharmacokinetic challenges inherent to this population.

Unlike the DOLPHIN study, where drug concentrations were measured at three predefined time points, TDM in our study was restricted to sparse sampling (predominantly a single trough concentration) driven entirely by standard clinical workflow and the strict need to minimize iatrogenic blood loss in neonates, rather than a protocolized research schedule. As increased sampling density is known to improve Bayesian exposure estimation, this difference may partially explain the absence of a statistically significant improvement in PD target attainment (Ewoldt et al., 2022).

Previous neonatal studies have reported more pronounced benefits of MIPD for vancomycin. In the prospective validation study by Kalamees et al., vancomycin dosing guided by a dedicated MIPD software significantly increased attainment of both trough concentration targets and AUC/MIC thresholds (Kalamees et al., 2024). While this multicenter design represents a methodological strength, the comparison of prospectively followed intervention patients with retrospectively evaluated controls introduces potential bias related to differences in patient characteristics, clinical practice, and data completeness.

Although the MIPD strategy resulted in a numerically higher target attainment for vancomycin compared to standard dosing, the overall success rate (16.7%) is notably lower than the 60%–80% attainment rates frequently reported in strictly controlled MIPD efficacy trials (Leroux et al., 2016; Kalamees et al., 2024; Van Wynsberge et al., 2024). This discrepancy directly reflects the pragmatic, real-world design of our study and the methodological constraints inherent to routine NICU care. First, our Bayesian forecasting relied predominantly on sparse, routine TDM sampling (typically a single trough concentration) rather than protocolized, multi-point pharmacokinetic sampling, which limits the precision of the Bayesian Maximum A Posteriori (MAP) algorithm. Second, to maintain a conservative safety profile in culture-negative sepsis, we assumed an empiric MIC of 1 mg/L; achieving an AUC/MIC ≥400 with an MIC of 1 mg/L inherently requires much higher absolute drug exposures compared to institutions that assume an MIC of 0.5 mg/L. Finally, our analysis strictly evaluated exposure attainment immediately following the first TDM-informed dose adjustment (T1). In real-world Bayesian dosing, target attainment is an iterative process that typically improves with subsequent sampling and sequential model updates.

Evidence from adult and pediatric populations further supports the clinical utility of Bayesian dosing approaches. Chai et al. demonstrated that Bayesian-guided dose adjustment using ID-ODS™ significantly improved therapeutic target attainment for vancomycin and other antimicrobials in critically ill adults with sub- or supratherapeutic drug levels (Chai et al., 2023). Similarly, in pediatric patients aged 2–16 years receiving various antimicrobials, a high prevalence of under- and overdosing was reported, emphasizing the need for MIPD to improve target attainment (Velarde-Salcedo et al., 2023).

Neonatal-specific PK variability remains a critical challenge. In a large multicenter PoppK study, Alsultan et al. demonstrated that very low birth weight neonates had a substantially lower probability of achieving vancomycin AUC/MIC >400 using standard dosing regimens, highlighting the inadequacy of current dosing recommendations for this population (Alsultan et al., 2023). These findings strongly align with our descriptive heatmap analyses, which demonstrated the severe inadequacy of standard static regimens, resulting in subtherapeutic vancomycin exposures (AUC/MIC <400) in 96.2% of our standard dosing cohort. In our cohort, subtherapeutic exposure predominated across all postnatal age and weight strata rather than being confined to any particular maturational subgroup, indicating that the shortfall of static dosing tables was not restricted to the most mature infants. Consequently, individualized dosing strategies via MIPD are particularly relevant in overcoming the extreme physiological and developmental variability that standard regimens fail to capture.

For amikacin, previous studies have also reported improved target attainment with model-based dosing. In line with this, our recent prospective feasibility study in critically ill pediatric patients demonstrated modest improvements in model fit and predictive performance with MIPD implementation, while also highlighting practical challenges related to real-world integration and variability in clinical conditions (Bayraktar et al., 2026). Smits et al. showed higher proportions of patients achieving both peak and trough targets without increased toxicity in the model-based dosing group compared with conventional regimens (Smits et al., 2015). In our study, for amikacin, target attainment did not differ significantly between groups, which may reflect the inherent limitations of our sparse, routine sampling approach. Clinically, our workflow prioritized patient safety by minimizing iatrogenic blood loss, which precluded the systematic measurement of peak concentrations. Consequently, the Cmax/MIC indices evaluated in this study were derived via Bayesian extrapolation rather than direct peak sampling. While this approach is pragmatic for routine NICU care, it introduces an inherent uncertainty in estimating Cmax-driven pharmacodynamic targets, which should be considered when interpreting our amikacin outcomes. This uncertainty is compounded by the fact that 26 of 53 first amikacin samples (49.1%) were below the limit of quantification. Importantly, the impact of a BLQ trough is not uniform across pharmacokinetic metrics. In a one-compartment framework, a BLQ trough constrains clearance only from below and is therefore weakly informative, so the individual posterior clearance is shrunk toward the population prior, whereas Cmax following a given dose is governed predominantly by the volume of distribution, which is weight-scaled and comparatively well identified. Cmax/MIC is therefore less sensitive to BLQ observations than clearance-driven metrics such as AUC or predicted trough. Consistent with this, a sensitivity analysis restricted to neonates with quantifiable first samples preserved both the direction and the magnitude of the between-group difference, and BLQ observations were numerically more frequent in the MIPD group, indicating that the higher attainment observed with MIPD is not an artefact of BLQ handling (Supplementary Table S4). This relative insensitivity to BLQ is, however, the counterpart of a more fundamental limitation: because a trough-only design carries little information about the volume of distribution, the derived Cmax remains close to the weight-scaled population prior in all neonates, not only in those with BLQ samples, and therefore carries the population between-subject variability in volume of distribution as irreducible uncertainty. Consistent with this, the classification proved sensitive to modest error in the estimated Cmax: the median neonate lay only 14.6% from a category boundary, and a 20% error in Cmax would reclassify up to 42% of the cohort, with the between-group difference reversing beyond a multiplicative error of 0.8–1.2 (Supplementary Table S6). The amikacin Cmax/MIC findings should therefore be read as an exploratory, category-level description of exposure rather than as a precise measure of peak-driven target attainment. A further and more specific concern is that the posterior was not meaningfully updated by the observation in half of the neonates with a sample below the quantification limit, in whom the model-predicted concentration remained an order of magnitude above the measured value and the platform classified the fit as poor. Because these neonates were preferentially classified as supratherapeutic, the amikacin supratherapeutic rate reported here is likely to be overstated. This misclassification was, however, distributed across both study phases and therefore largely non-differential, and the between-group comparison was preserved when observations below the quantification limit were excluded (Supplementary Table S4).

Importantly, unlike many previous studies that assumed a fixed MIC of 1 mg/L when calculating PD indices, our study incorporated pathogen-specific or ECOFF MIC values. This approach better accounts for interspecies variability in susceptibility and improves the clinical relevance of PK/PD assessments (Van Wynsberge et al., 2024). Nevertheless, because most neonates were treated empirically, the great majority of PK/PD evaluations still rested on an assumed MIC, and our sensitivity analysis shows that this assumption dominates the absolute attainment rates. Halving the assumed vancomycin MIC to 0.5 mg/L raised therapeutic attainment from 3.8% to 50.0% under standard dosing and from 16.7% to 40.0% under MIPD, while doubling it to 2 mg/L rendered the target unattainable in every neonate. The ordering of the therapeutic band is therefore not robust to this assumption, and the reversal observed at 0.5 mg/L reflects a shift of the MIPD group into the supratherapeutic band rather than a loss of exposure. Across the MIC assumptions at which the target was attainable, the MIPD group was less frequently underexposed, as expected given that the MIC assumption rescales the denominator of the PK/PD index without altering the absolute exposure achieved. Absolute attainment rates reported here should accordingly not be compared directly with studies adopting a different MIC convention.

Beyond evaluating target attainment, assessing the concordance between observed concentrations and individual Bayesian estimates is essential. The population PK models utilized in our study (Frymoyer et al. for vancomycin and Illamola et al. for amikacin) were originally derived from and validated in large neonatal cohorts with demographics comparable to our NICU population, supporting their baseline validity. As detailed in our results, posterior model concordance was nonetheless limited for vancomycin and modest for amikacin. Such deviations are consistent with previous reports (Hughes et al., 2023; Bayraktar et al., 2026). Critically ill neonates undergo rapid, dynamic changes in renal maturation and fluid status that challenge even the most robust Bayesian priors (Smits et al., 2013; Allegaert et al., 2017). Because our error metrics were themselves derived from posterior estimates that already incorporate the measured concentration, they represent a favorable bound on model agreement; the concordance achievable from population priors alone would be lower. This constrains the confidence that can be placed in model-derived exposure estimates in this population.

### 4.2 Clinical outcomes

Although treatment duration represents a healthcare utilization metric rather than a direct clinical outcome, it was directly compared between groups in our study to evaluate antimicrobial exposure, with no statistically significant difference observed (Ewoldt et al., 2022). However, the treatment-duration-to-hospital-length-of-stay ratio (DOT/LOS) was significantly lower in the MIPD group, which may reflect differences in antimicrobial utilization patterns between groups. Length of hospital stay and mortality did not differ significantly between groups, consistent with previous adult and neonatal studies (Ewoldt et al., 2022; Hall et al., 2024).

Other studies have reported improvements in surrogate clinical outcomes following MIPD implementation. Gomez et al. demonstrated shorter time to blood culture negativity and reduced need for antibiotic modification in neonates after implementation of a Bayesian dosing protocol, without increased nephrotoxicity (Gomez et al., 2022). However, similar to our findings, treatment duration and length of stay were not significantly affected.

### 4.3 Biomarkers of treatment response

Changes in inflammatory biomarkers, particularly CRP, did not differ significantly between groups, consistent with findings from the DOLPHIN study (Ewoldt et al., 2022). Procalcitonin, a promising biomarker for monitoring treatment response, could not be fully evaluated due to limited measurements in the intervention group. Previous analyses suggest that changes in procalcitonin may be associated with antimicrobial exposure, although evidence remains limited (Saleh et al., 2019; Drager et al., 2024).

Notably, changes in SNAPPE-II scores differed significantly between groups, with a greater median reduction observed in the standard care cohort. However, this finding must be interpreted with extreme caution. SNAPPE-II is primarily validated for illness severity stratification at admission and lacks robustness for longitudinal monitoring. Because baseline SNAPPE-II scores were comparable between groups (Table 1), this difference cannot be explained by a measured imbalance in illness severity at the time of antimicrobial initiation. We additionally examined whether the between-phase imbalance in daily fluid intake could account for it and found no association between daily fluid intake and ΔSNAPPE-II, either overall (Spearman’s rho = 0.124, p = 0.354) or within either study phase. The most plausible interpretation is therefore residual unmeasured confounding inherent to a non-randomized before-after design, rather than a genuine adverse effect or inferior performance of the MIPD strategy, and this finding should be regarded as hypothesis-generating only. Given that higher SNAPPE-II scores indicate greater illness severity and mortality risk, negative ΔSNAPPE-II values reflect clinical improvement. Consequently, further prospective, randomized studies are required to clarify the role of serial SNAPPE-II assessments in evaluating therapeutic response in this population.

### 4.4 Safety and adverse effects

No statistically significant differences were detected between groups regarding nephrotoxicity or ototoxicity. The precision of these comparisons was, however, very low: the risk difference was +3.1% for acute kidney injury (95% CI −13.3% to +19.5%) and −0.9% for ototoxicity (95% CI −17.4% to +16.9%), so the data remain compatible with either a substantial reduction or a substantial increase in both outcomes. These findings are consistent with previous pediatric and neonatal studies reporting that MIPD improves target attainment without increasing adverse event rates (Kalamees et al., 2024; Stokes et al., 2025). However, these reassuring safety observations should be interpreted with caution. The small sample size of our cohort limits the statistical power to identify rare drug-related adverse events. Furthermore, while BERA screening is a standard clinical practice, it may lack the sensitivity required to detect subtle, subclinical, or progressive drug-related ototoxicity in the neonatal intensive care setting. While some studies have reported reduced nephrotoxicity with MIPD-guided vancomycin dosing (Leroux et al., 2016), our study was not powered to evaluate this and we therefore draw no conclusion about the comparative toxicity of the two strategies.

Recent meta-analyses evaluating individualized antimicrobial dosing corroborate our safety findings; they demonstrate that MIPD improves target attainment without increasing the risk of adverse events, although they have not definitively shown statistically significant reductions in overall mortality or length of stay (Sanz-Codina et al., 2023; Yalcin et al., 2025). Our study likewise observed favourable exposure trends with no detected increase in nephrotoxicity or other adverse outcomes, but the number of events was too small for this to be interpreted as evidence of safety.

### 4.5 Fluid balance and drug clearance

Consistent with previous studies, body weight, PMA, and SCr were important determinants of vancomycin and amikacin clearance (Dao et al., 2020; Lee et al., 2021; Zhao et al., 2025). In our unadjusted analysis, absolute daily fluid administration (mL/day) showed a positive correlation with estimated drug clearance; however, we acknowledge that this finding is highly susceptible to confounding bias.

Indeed, fluid administration is closely linked to body size and maturational status, as larger and more mature neonates typically receive greater absolute fluid volumes and exhibit higher drug clearance. Therefore, the observed association may partly reflect underlying differences in body weight and developmental physiology rather than an independent effect of fluid administration *per se*. This is also relevant to the interpretation of our between-group comparisons, because daily fluid intake was higher during the intervention phase than during the observational phase (382 vs. 240 mL/day, p = 0.024), a difference that persisted after normalization to body weight (147.6 vs. 120.7 mL/kg/day, p = 0.035) and therefore reflects a genuine change in fluid management between the two periods rather than a difference in body size alone. Two considerations limit its impact. First, the imbalance was not associated with the clinical endpoints: daily fluid intake showed no correlation with ΔSNAPPE-II (rho = 0.124, p = 0.354), the DOT/LOS ratio (rho = 0.028, p = 0.837) or length of stay (rho = 0.042, p = 0.753), estimated glomerular filtration rate did not differ between phases (Table 3), and diuretic use was comparable (16.7% vs. 29.0%, p = 0.363). Second, with respect to drug exposure the direction of any bias is conservative: because higher fluid intake was associated with higher estimated clearance and therefore with lower exposure, the MIPD group attained higher PK/PD targets despite a covariate distribution expected to work against it. It should also be noted that daily fluid intake was recorded during therapy rather than at enrolment, and thus represents a time-varying feature of supportive care in each study period rather than a baseline characteristic. Residual confounding from this imbalance cannot be fully excluded. These findings suggest that dynamic clinical variables may be relevant for dose individualization.

### 4.6 Limitations

This study has several limitations. The lack of integration between the MIPD software and the hospital electronic health record necessitated manual data entry, increasing workload and potential for error. Limited availability and delayed reporting of TDM results restricted sampling frequency and timely dose adjustment. Observed concentration comparisons between groups are limited by non-standardized sampling times and should be interpreted with caution. Because no comparable neonatal MIPD outcome data were available, no *a priori* sample size calculation was performed, and the study was consequently underpowered for its primary comparisons: with 26 and 30 evaluable neonates for vancomycin, the observed difference in therapeutic attainment lies well below the effect size detectable at conventional power. The non-significant between-group comparisons should therefore be read as uninformative rather than as evidence of no difference. The small sample size and single-center design limit generalizability, and the lack of comprehensive model options for certain clinical scenarios (e.g., specific concomitant medications or dialysis parameters) may have constrained model concordance. Although the cohort was not restricted by body weight and included neonates with birth weights as low as 630 g, extremely low birth weight neonates remain under-represented. The small number of patients in this stratum precludes reliable subgroup inference, and our findings should be applied with particular caution in this group, in whom pharmacokinetic variability and the frequency of unquantifiable concentrations are greatest. A structural limitation of the design is that Bayesian exposure simulations under revised regimens were available only for the intervention phase, since the platform was not in use during the observational phase. The relative effectiveness of Bayesian dose revision versus conventional linear scaling therefore could not be compared directly, and establishing this would require randomisation or parallel prospective simulation alongside standard care. Additionally, exploratory pharmacometric analyses were not primary endpoints and should be interpreted cautiously. A fundamental limitation of the MIPD approach itself is its inherent reliance on the overall predictive performance of the underlying population models. As demonstrated by our error metrics, posterior model concordance remained limited, particularly for amikacin; because these metrics were computed against estimates that already incorporate the measured concentration, they bound model agreement optimistically and do not quantify prospective predictive performance. When a model’s baseline concordance is modest, the Bayesian algorithm’s ability to safely optimize doses is significantly constrained unless it is supported by rich, multi-point pharmacokinetic sampling, a practice that is often clinically prohibitive in neonates due to blood conservation protocols. Relatedly, population (*a priori*) concentration predictions were not extracted for comparison, so the accuracy with which these models would have predicted concentrations before any sample was available was not quantified. We have previously reported such *a priori* versus *a posteriori* comparison in a paediatric cohort using the same platform (Bayraktar et al., 2026). It was not replicated here because the observational phase was reconstructed retrospectively from routine records, so *a priori* predictions generated after the fact would not correspond to the information actually available at the time of dosing. This is a meaningful gap, because in the intervention phase the initial dose was selected from *a priori* model output, and the adequacy of that first recommendation therefore remains unevaluated. Formal assessment of *a priori* predictive performance should be a priority in future neonatal MIPD studies. Furthermore, the predominance of poor to moderate model fit may limit the reliability of the estimated individual pharmacokinetic parameters, which should be considered when interpreting the observed associations between pharmacokinetic estimates and clinical covariates. In the absence of pathogen-specific MIC data, default MIC values were necessarily assumed for PK/PD analyses, which may have limited the precision of target attainment estimates. Also, this study was not randomized and followed a before-after design, with observational and intervention phases conducted sequentially over time. Therefore, the possibility of temporal bias and secular trends cannot be excluded, as changes in clinical practice, evolving case-mix, infection epidemiology, and growing experience with the MIPD approach may have influenced the observed findings independently of the intervention itself. One such difference was observed directly in our data: daily fluid intake, both absolute and normalized to body weight, was higher during the intervention phase, and although it was not associated with any of the clinical endpoints, it illustrates the vulnerability of sequential non-randomized cohorts to unmeasured changes in supportive care.

Furthermore, the high number of ‘zero’ values in initial amikacin measurements which clinically represent trough concentrations falling BLQ is a significant methodological limitation. This frequent finding is primarily a consequence of routine clinical sampling times (often drawn >96 h post-dose, well beyond the drug’s elimination half-life) rather than an insensitivity of the LC-MS/MS bioanalytical assay itself. The presence of these BLQ values not only precluded the use of standard percentage-based error metrics due to mathematical constraints, but it also limits the Bayesian algorithm’s ability to precisely estimate true drug clearance and may introduce both imprecision and directional bias into the individual estimates: in neonates whose posterior did not accommodate the measured value, exposure was systematically overestimated, and the individual clearance values used in our exploratory covariate analyses (Table 4; Figure 5) should be interpreted with this in mind. This challenge underscores the critical need for highly sensitive bioanalytical assays and advanced censored-data methodologies in future neonatal aminoglycoside modeling. Because the platform conditions on entered concentrations as observed point values rather than applying likelihood-based censored-data methods, the censored nature of these observations could not be formally incorporated; the sensitivity analysis restricted to quantifiable samples (Supplementary Table S4) was therefore used to assess the robustness of the amikacin findings. A formal confidence interval for each neonate’s estimated Cmax could not be derived, as the platform does not export posterior standard errors for individual pharmacokinetic parameters; direct peak sampling would be required to establish this endpoint reliably.

A further limitation relates to the definition of AKI. Due to the lack of consistent urine output measurements, AKI was defined using only the serum creatinine component of the KDIGO criteria. This approach may have underestimated the true incidence of AKI, as urine output is an integral part of the KDIGO definition. Additionally, interpretation of SCr in neonates is complicated by physiological postnatal changes and the influence of maternal creatinine during the first days of life. Therefore, AKI findings should be interpreted with caution.

## 5 Conclusion

Standard dosing strategies may lead to suboptimal exposure in neonates due to marked PK variability. MIPD represents a clinically feasible approach to individualize vancomycin and amikacin therapy by integrating patient-specific data with TDM. In this study, MIPD-based dosing recommendations were consistently utilized by the NICU physicians as part of the clinical workflow, and our findings were associated with numerically but not statistically higher target attainment, without a detected increase in adverse events. However, given the exploratory design and limited sample size, these findings should be interpreted cautiously, and further adequately powered prospective studies are required to determine the clinical impact of MIPD in critically ill neonates.

## Data Availability

The datasets generated and/or analysed during the current study are not publicly available due to ethical restrictions. Requests to access these datasets should be directed to the corresponding author.
