# Supplementary material for: Model-informed precision dosing of vancomycin and amikacin in critically ill neonates: a pre- and post-intervention study
Source: Front Pharmacol. 2026 Oct 16;17:1985125. doi: 10.3389/fphar.2026.1985125 (PMC13719479; doi:10.3389/fphar.2026.1985125)
Supplement: Supplementary file 1 [file Supplementaryfile1.docx]

**Performance Measurements**

Posterior model concordance was evaluated by calculating the mean prediction error (MPE) and relative root mean squared error (rRMSE) using R software (R Foundation for Statistical Computing, Vienna, Austria) along with the dplyr and Metrics packages. In accordance with established pharmacometric guidelines, concordance was deemed clinically acceptable if the relative bias (rBias) was within ±20% and precision metrics (such as MAPE or rRMSE) were ≤30% [1]. Model performance was assessed using both mean-based and median-based error metrics.

$$Mean Absolute Error \left( \mathrm{MAE} \right)=\frac{1}{n}\sum_{i=1}^{n} \left| \mathrm{predicted}_{i}-\mathrm{observed}_{i} \right|$$

$$Mean Absolute Percentage Error \left( \mathrm{MAPE} \right)=\frac{1}{n}\sum_{i=1}^{n} \left| \frac{\mathrm{predicted}_{i}-\mathrm{observed}_{i}}{\mathrm{observed}_{i}} \right|$$

$$Relative Bias (rBias)=\frac{\frac{1}{n}\sum_{i=1}^{n} (\mathrm{predicted}_{i}-\mathrm{observed}_{i})}{\frac{1}{n}\sum_{i=1}^{n} \mathrm{observed}_{i}}\times100$$

$$Root Mean Squared Error (RMSE) =\sqrt{\frac{1}{n}\times\sum_{i=1}^{n} {(\mathrm{predicted}_{i}-\mathrm{observed}_{i})}^{2}}$$

$$Relative Root Mean Squared Error \left( \mathrm{rRMSE} \right) =\sqrt{\frac{1}{n}\times\sum_{i=1}^{n} \frac{{(\mathrm{predicted}_{i}-\mathrm{observed}_{i})}^{2}}{{{((observed}_{i}+\mathrm{predicted}_{i})/2)}^{2}}\times100}$$

$$Median Absolute Error \left( \mathrm{MdAE} \right)=median(\left| \mathrm{predicted}_{i}-\mathrm{observed}_{i} \right|)$$

$$Median Absolute Percentage Error \left( \mathrm{MdAPE} \right)=median\left( \left| \frac{\mathrm{predicted}_{i}{-observed}_{i}}{\mathrm{observed}_{i}} \right| \right)\times100$$

$$Median Error \left( \mathrm{MdE} \right) or Robust bias (robBIAS)=median(\mathrm{predicted}_{i}-\mathrm{observed}_{i})$$

$$Root Median Squared Error (RMdSE) =\sqrt{\mathrm{median}{(\mathrm{predicted}_{i}-\mathrm{observed}_{i})}^{2}}$$

$$Relative Root Median Squared Error (rRMdSE)=\frac{\sqrt{median({(\mathrm{predicted}_{i}-\mathrm{observed}_{i})}^{2})}}{median(\mathrm{observed}_{i})}\times100$$

**Posterior Model Concordance and Error Metrics**

Posterior model concordance for vancomycin and amikacin concentrations was evaluated using statistical error metrics. Measured and predicted vancomycin concentrations across all sampling points for the overall pooled cohort are presented in Table S1, while the mean- and median-based error metrics reflecting the posterior model concordance of the vancomycin model are shown in Table S2. For vancomycin, the MAE for the entire sample was 5.47 mg/L, indicating that the model deviated from the measured concentrations by approximately 5 to 5.5 mg/L on average. Overall MAPE for the pooled cohort was 128.1%, and overall rBias was 12.5%. Although error rates appeared relatively high when assessed using mean-based metrics, median-based measures (MdAE 4.06 mg/L) were lower, suggesting that a limited number of outliers may have inflated the mean error estimates.

**Table S1.** Distribution of measured and posterior-predicted vancomycin concentrations across all sampling points (pooled)

|  | **Total** | | | |
| --- | --- | --- | --- | --- |
|  | **n** | **Mean** | **Min** | **Max** |
| **Measured vancomycin concentration (mg/L)** | 62 | 10.83 | 0.33 | 60.50 |
| **Posterior-predicted vancomycin concentration (mg/L)** | 62 | 12.18 | 0.28 | 38.38 |

**Table S2.** Mean- and median-based error metrics for the posterior model concordance of the vancomycin model

|  | **Mean -based metrics** | | | | |  | **Median -based metrics** | | | |
| --- | --- | --- | --- | --- | --- | --- | --- | --- | --- | --- |
|  | **MAE** | **MAPE** | **rBias (%)** | **RMSE** | **rRMSE (%)** | **MdAE** | **MdAPE** | **MdE** | **RMdSE** | **rRMdSE (%)** |
| Overall  (n = 62) | 5.47 | 128.11 | 12.49 | 7.28 | 72.23 | 4.06 | 37.66 | 1.54 | 4.06 | 57.96 |

MAE, mean absolute error; MAPE, mean absolute percentage error; rBias, relative bias; RMSE, root mean squared error; rRMSE, relative root mean squared error; MdAE, median absolute error; MdAPE, median absolute percentage error; MdE, median error (robust bias); RMdSE, root median squared error; rRMdSE, relative root median squared error.

In addition to mean-based metrics, median-based errors were evaluated to minimize the influence of extreme outliers. For the overall pooled cohort, the median prediction error (MdE) was 1.54 mg/L, indicating only a slight tendency toward overestimation, while the median absolute percentage error (MdAPE) was 37.66%. Due to the presence of zero values in first amikacin concentration measurements and the inability to include zero values in the denominator, these metrics were not calculated for amikacin.

Measured and model-predicted amikacin concentrations are summarized in Table S3. Error metrics could not be calculated for the second drug concentration measurements due to the very limited number of observations. For the first amikacin concentration measurements, the presence of a large number of zero values and a median of zero precluded calculation of MAPE, rBias, and rRMdSE.

In the overall sample for amikacin, the mean absolute error (MAE) was 8.96 and the root mean square error (RMSE) was 13.87. Median-based metrics (MdAE = 1.31) indicated a low typical prediction error of the model.

**Table S3.** Distribution of measured and posterior-predicted amikacin concentrations across all sampling points (pooled)

|  | **Total** | | | |
| --- | --- | --- | --- | --- |
|  | **n** | **Mean** | **Min** | **Max** |
| Measured amikacin concentration (mg/L) | 58 | 0.47 | 0.00 | 4.00 |
| Posterior-predicted amikacin concentration (mg/L) | 58 | 9.43 | 0.46 | 41.76 |

Because 26 of 53 first amikacin samples (49.1%) were below the limit of quantification, a sensitivity analysis was performed restricting the Cmax/MIC comparison to neonates with a quantifiable first concentration. The BLQ rate did not differ significantly between groups (standard dosing 11/26, 42.3%; MIPD 15/27, 55.6%; Fisher's exact p = 0.414). The direction and magnitude of the between-group difference in target attainment were preserved in the restricted analysis (Table S4).

**Table S4.** Sensitivity analysis: amikacin Cmax/MIC target attainment before the first dose adjustment, full cohort versus neonates with a quantifiable first (T1) concentration

| **Cmax/MIC category** | **Full cohort-Standard (n=26)** | **Full cohort-MIPD (n=27)** | **Quantifiable T1-Standard (n=15)** | **Quantifiable T1-MIPD (n=12)** |
| --- | --- | --- | --- | --- |
| Subtherapeutic (<8) | 8 (30.8%) | 5 (18.5%) | 4 (26.7%) | 2 (16.7%) |
| Therapeutic (8 to <11) | 5 (19.2%) | 10 (37.0%) | 3 (20.0%) | 5 (41.7%) |
| Supratherapeutic (≥11) | 13 (50.0%) | 12 (44.4%) | 8 (53.3%) | 5 (41.7%) |
| p-value | 0.304 ᵃ | | 0.599 ᵇ | |
| Median Cmax/MIC (Q1-Q3) | 11.4 (6.3-12.8) | 10.4 (8.8-12.7) | 11.9 (7.2-14.2) | 10.4 (9.3-11.8) |
| p-value | 0.852 ᶜ | | 0.678 ᶜ | |

ᵃ Pearson chi-square test (all expected counts ≥5). ᵇ Fisher–Freeman–Halton exact test (4 of 6 expected counts <5). ᶜ Mann–Whitney U test. BLQ, below the limit of quantification. Quantifiable T1 = neonates whose first amikacin concentration was at or above the assay quantification limit (n=27 of 53; 26 samples, 49.1%, were LLOQ).

In a MIC-independent analysis of absolute exposure restricted to neonates evaluated against the empiric MIC (n=48), median AUC0-24h was 237 mg·h/L (Q1-Q3, 197-290) under standard dosing and 233 mg·h/L (180-430) under MIPD (p = 0.631); an AUC0-24h of at least 400 mg·h/L was reached in 1/20 (5.0%) and 8/28 (28.6%) of neonates, respectively (p = 0.061) (Table S5).

**Table S5.** Sensitivity analysis of PK/PD target attainment across alternative empiric MIC assumptions (pathogen-specific ECOFF values held constant)

| **Agent** | **Assumed MIC** | **Standard: sub / therapeutic / supra** | **MIPD: sub / therapeutic / supra** | **Therapeutic (%)** | **At or above target (%)** | **p** |
| --- | --- | --- | --- | --- | --- | --- |
| Vancomycin (n=56) | 0.5 mg/L | 11 / 13 / 2 | 10 / 12 / 8 | 50.0 vs 40.0 | 57.7 vs 66.7 | 0.215 |
|  | **1 mg/L (base case)** | **25 / 1 / 0** | **22 / 5 / 3** | **3.8 vs 16.7** | **3.8 vs 26.7** | **0.068** |
|  | 2 mg/L | 26 / 0 / 0 | 30 / 0 / 0 | 0 vs 0 | 0 vs 0 | 1.000 |
| Amikacin (n=53) | 2 mg/L | 3 / 2 / 21 | 1 / 3 / 23 | 7.7 vs 11.1 | 88.5 vs 96.3 | 0.660 |
|  | **4 mg/L (base case)** | **8 / 5 / 13** | **5 / 10 / 12** | **19.2 vs 37.0** | **69.2 vs 81.5** | **0.336** |
|  | 8 mg/L | 24 / 2 / 0 | 26 / 1 / 0 | 7.7 vs 3.7 | 7.7 vs 3.7 | 0.610 |

Standard dosing n=26 for both agents; MIPD n=30 for vancomycin and n=27 for amikacin. Vancomycin categories: subtherapeutic AUC/MIC <400, therapeutic 400-700, supratherapeutic >700. Amikacin categories: subtherapeutic Cmax/MIC <8, therapeutic 8 to <11, supratherapeutic ≥11. "At or above target" = therapeutic plus supratherapeutic. p-values from the Fisher–Freeman–Halton exact test applied uniformly across scenarios; the base-case amikacin comparison reported in Table 2 (p = 0.304) used the Pearson chi-square test, as all expected frequencies exceeded five.

Because amikacin Cmax was extrapolated from predominantly trough samples rather than measured directly, the stability of the Cmax/MIC classification was examined. The therapeutic band (Cmax/MIC 8 to <11) spans 37.5% of its lower bound, and the median neonate lay 14.6% from the nearest boundary (18/53, 34.0% within 10%; 29/53, 54.7% within 20%). Boundary proximity was comparable in neonates with a BLQ first sample (median 14.5%) and in those with a quantifiable sample (median 15.6%), indicating that this fragility reflects the narrow therapeutic band rather than the BLQ observations specifically (Table S6).

**Table S6.** Robustness of the amikacin Cmax/MIC classification to systematic error in the extrapolated Cmax

| **Multiplicative error applied to Cmax** | **Standard (n=26) sub / therapeutic / supra** | **MIPD (n=27) sub / therapeutic / supra** | **Therapeutic, % (Standard vs MIPD)** | **Neonates reclassified vs base case** |
| --- | --- | --- | --- | --- |
| ×0.7 | 13 / 10 / 3 | 18 / 8 / 1 | 38.5 vs 29.6 | 36/53 (67.9%) |
| ×0.8 | 11 / 9 / 6 | 11 / 10 / 6 | 34.6 vs 37.0 | 22/53 (41.5%) |
| ×0.9 | 9 / 7 / 10 | 8 / 11 / 8 | 26.9 vs 40.7 | 11/53 (20.8%) |
| **×1.0 (base case)** | **8 / 5 / 13** | **5 / 10 / 12** | **19.2 vs 37.0** | — |
| ×1.1 | 8 / 3 / 15 | 4 / 7 / 16 | 11.5 vs 25.9 | 7/53 (13.2%) |
| ×1.2 | 8 / 1 / 17 | 4 / 5 / 18 | 3.8 vs 18.5 | 11/53 (20.8%) |
| ×1.3 | 6 / 3 / 17 | 4 / 1 / 22 | 11.5 vs 3.7 | 17/53 (32.1%) |

Categories: subtherapeutic Cmax/MIC <8, therapeutic 8 to <11, supratherapeutic ≥11. The multiplicative factors are illustrative bounds used to probe the stability of the classification and are not derived from the model's error structure, as the platform does not export posterior standard errors for individual parameters. The direction of the between-group difference in therapeutic attainment is preserved between ×0.8 and ×1.2 and reverses at ×0.7 and ×1.3.

Furthermore, visual concordance is illustrated in Figure S1, which displays the goodness-of-fit plot comparing the measured (observed) concentrations with individual Bayesian posterior predictions for both agents.

**Figure S1.** Goodness-of-fit (GOF) plot comparing observed concentrations with individual Bayesian posterior (a posteriori) predictions for vancomycin and amikacin. Because the posterior estimates incorporate the observed concentration, this plot depicts posterior concordance and not prospective predictive performance. The solid red line represents the linear regression fit with the 95% confidence interval (shaded area), and the dashed black line represents the line of identity (y = x).

GOF = goodness-of-fit; mg/L = milligrams per liter; R² = coefficient of determination.

**References**

1.	Yano I, Beal SL, Sheiner LB. The need for mixed-effects modeling with population dichotomous data. J Pharmacokinet Pharmacodyn. 2001 Aug;28(4):389-412.
